# Molecular prevalence, genomic characterization, and zoonotic potential of novel paramyxovirus and hepacivirus in *Alexandromys fortis*, Republic of Korea

**DOI:** 10.1186/s13567-026-01777-z

**Published:** 2026-05-28

**Authors:** Haryo Seno Pangestu, Intae Yang, Augustine Natasha, Shivani Rajoriya, Hennisa Hennisa, Jieun Park, Kyungmin Park, Jongwoo Kim, Seong-Gyu Kim, Terry A. Klein, Heung Chul Kim, Yeonsu Oh, Jin-Won Song, Won-Keun Kim

**Affiliations:** 1 Department of Microbiology, College of Medicine, Hallym University, Chuncheon, 24252 Republic of Korea; 2 College of Medicine, Hallym University, Chuncheon, 24252 Republic of Korea; 3 Department of Microbiology, Korea University College of Medicine, Seoul, 02841 Republic of Korea; 4 Institute for Viral Diseases, Korea University College of Medicine, Seoul, 02841 Republic of Korea; 5 BK21 Graduate Program, Department of Biomedical Sciences, Korea University College of Medicine, Seoul, Republic of Korea; 6 Force Health Protection and Preventive Medicine, 65th Medical Brigade/US Army MEDDAC-Korea, Unit 15281, APO, AP, 96271-5281 USA; 7 College of Veterinary Medicine and Institute of Veterinary Science, Kangwon National University, Chuncheon, Republic of Korea; 8 Institute of Medical Science, College of Medicine, Hallym University, Chuncheon, 24252 Republic of Korea; 9 Present Address: PSC 450, APO AP, PSC 450, Box 75R, APO, AP, 96206 USA; 10 Present Address: Ucarlix, 169-23, Gasan Digital 2-ro, Geumcheon-gu, Seoul, 08504 Republic of Korea

**Keywords:** *Alexandromys fortis*, rodent, reed vole, metagenomic sequencing, *Paramyxoviridae*, *Jeilongvirus*, *Flaviviridae*, *Hepacivirus*, zoonotic potential

## Abstract

Rodents are substantial reservoirs of zoonotic viruses with regular human exposure restricted to a limited number of species. Numerous rodent species have been shown to harbor emerging viruses, including paramyxoviruses and hepaciviruses. Reed voles (*Alexandromys fortis*), a rodent species that inhabits grasslands and riparian environments throughout East Asia, remain poorly characterized in terms of their viral diversity. In this study, 258 *A*. *fortis* specimens collected from rural areas in Gyeonggi Province, Republic of Korea (ROK) were screened for paramyxoviruses and subjected to metagenomic next-generation sequencing. Genome characterization, phylogenetic and cophylogenetic assessments, and prediction of signal peptidase cleavage sites were performed to analyze the molecular features of the identified viruses. Zoonotic potential was evaluated using a genome-based machine-learning model. A nearly complete genome of a novel paramyxovirus, designated as Pyeongtaek Alexandromys paramyxovirus (PyAPV), was identified in six *A*. *fortis* specimens, with all sequences clustering within the genus *Jeilongvirus*. A nearly complete genome of a rodent-associated hepacivirus was also obtained from four specimens and classified as a distinct lineage within the species *Hepacivirus J*. These findings demonstrate the role of *A*. *fortis* as a natural reservoir of emerging viruses and expand current knowledge of rodent-associated viral diversity in the ROK.

**Supplementary Information:**

The online version contains supplementary material available at 10.1186/s13567-026-01777-z.

## Introduction

Emerging viral infections represent a persistent challenge for animal health, livestock production, and public health systems [1]. These infections originate from established virus–host associations in wildlife, following increased contact between reservoir hosts and humans or domestic animals [2]. Rodents play an important role in this process because several species frequently occur in agricultural, peri-urban, and rural environments [3, 4]. Viruses from the families *Paramyxoviridae*, *Flaviviridae*, *Hantaviridae*, and others have been repeatedly associated with infections in domestic animals, highlighting the importance of understanding their circulation in wildlife reservoirs [5–7].

The family *Paramyxoviridae* is a group of enveloped, nonsegmented, negative-sense RNA viruses classified into four subfamilies and 23 genera [8]. These viruses infect a wide range of vertebrate hosts and generally exhibit strong host specificity, with documented interspecies transmission events limited to a small number of genera [9]. Recent metagenomic studies have identified numerous novel paramyxoviruses in rodents, including members of the genus *Jeilongvirus* [10, 11]. Jeilongviruses are distinguished from other paramyxoviruses by the presence of a transmembrane (*TM*) gene in all members and a small hydrophobic (*SH*) gene in certain species, both located between the fusion (*F*) and receptor-binding protein (*RBP*) genes [12]. Notably, paramyxoviruses from the genera *Parahenipavirus* and *Jeilongvirus* have been reported in small mammals in the Republic of Korea (ROK) [13–15]. Although zoonotic transmission is well-documented for the genus *Henipavirus*, the growing detection of diverse rodent-associated paramyxoviruses, including *Jeilongvirus*, highlights the importance of assessing circulation in wildlife reservoirs and potential relevance to veterinary health [16].

The family *Flaviviridae* includes numerous important pathogens of animals and humans, and within this family the genus *Hepacivirus* has expanded considerably with the discovery of novel species in multiple mammalian hosts [17, 18]. Rodent hepaciviruses (RHVs) represent the most genetically diverse clade, with species reported from the deer mouse (*Peromyscus maniculatus*) with *Hepacivirus peromysci* (Hepacivirus E), Norway rat (*Rattus norvegicus*) with *Hepacivirus ratti* (Hepacivirus G) and *Hepacivirus norvegici* (Hepacivirus H), four-striped grass mouse (*Rhabdomys pumilio*) with *Hepacivirus rhabdomysis* (Hepacivirus I), and bank vole (*Myodes glareolus*) with *Hepacivirus myodae* (Hepacivirus F) and *Hepacivirus glareoli* (Hepacivirus J) [19–21]. RHVs exhibit the greatest genetic diversity among mammalian hepaciviruses and are hypothesized to be the primary source of hepaciviruses detected in mammals [22]. Beyond rodents, hepaciviruses have also been identified in horses and nonhuman primates, highlighting their capacity for cross-species infection and their relevance to veterinary medicine [23].

Among the various rodent species, the reed vole (*Alexandromys fortis*, formerly *Microtus fortis*) is widely distributed throughout northern and central Eurasia, inhabiting wetlands and grasslands that often overlap with agricultural landscapes [24]. This species has previously been identified as a host of hantaviruses in China and Russia, highlighting its potential relevance to zoonotic virus transmission [25, 26]. In the ROK, *A*. *fortis* has been reported to be seropositive for *Hantaan virus* (HTNV) at military training and rice-farming sites in Gyeonggi Province [27, 28]. In addition to hantaviruses, *A*. *fortis* was previously shown to be positive for a rodent-associated paramyxovirus, suggesting that this species may harbor a broader spectrum of viral taxa than currently recognized [29]. Understanding the viruses associated with *A*. *fortis* may offer insights into the ecological and evolutionary dynamics of emerging pathogens and their potential veterinary relevance.

Given the detection of multiple virus families in *A*. *fortis*, this study aimed to investigate paramyxoviruses and to examine any additional viral diversity potentially present in this species. A total of 258 *A*. *fortis* specimens were collected and examined from rural environments in the ROK between 2001 and 2009. Molecular screening and metagenomic next-generation sequencing using an unbiased taxonomic classification workflow yielded near-complete genomes of a novel paramyxovirus belonging to the genus *Jeilongvirus* and a genetically distinct hepacivirus within the species *Hepacivirus J*. These viruses formed distinct genetic lineages and exhibited coevolutionary patterns with their rodent host. Genome characterization, phylogenetic and cophylogenetic analyses, and signal peptidase cleavage site prediction were performed to analyze these viral genomic features, and a machine-learning model was applied to evaluate predicted zoonotic potential. These findings expand the knowledge of viruses associated with *A*. *fortis* and provide new insights into rodent-borne viral diversity at the wildlife–livestock interface.

## Materials and methods

### Ethics statement

The animal trapping procedure was approved by the US Forces Korea (USFK) in accordance with USFK Regulation 40–1 “Prevention, Surveillance, and Treatment of Hemorrhagic Fever with Renal Syndrome.” All procedures and handling of animals were conducted according to a protocol approved by the Korea University Institutional Animal Care and Use Committee (KUIACUC, #2010–212).

### Animal trapping and organ sampling

Small mammals were trapped between 2001 and 2009 in four rural localities in Gyeonggi Province (Paju, Pyeongtaek, Pocheon, and Yeoncheon) using Sherman traps (8 × 9 × 23 cm; H. B. Sherman, Tallahassee, FL, USA). Collection sites included agricultural fields, adjacent grasslands, and riparian zones near irrigation channels. Traps were placed at intervals of 3–4 m and inspected early the next morning for 1–2 consecutive days. Live animals were euthanized using compressed carbon dioxide in a dedicated chamber, following the American Veterinary Medical Association (AVMA) guidelines with death confirmed by the absence of heartbeat and corneal reflex [30]. Rodents were identified to the species level on the basis of their physical characteristics [31]. A total of 258 *A*. *fortis* specimens were necropsied and kidney tissues were obtained under aseptic conditions. All samples were preserved at –80 °C until subsequent analysis.

### Genetic identification of hosts by mitochondrial DNA (mtDNA) analysis

Specimens identified as *A. fortis *based on field morphological characteristics were confirmed by Sanger sequencing of the mtDNA *cytb* gene. Total RNA was extracted from kidney tissues using TRIzol™ Reagent (Thermo Fisher Scientific, Cat# 15596018, Waltham, MA, USA). Residual host genomic DNA present in the RNA preparation was used as the template for polymerase chain reaction (PCR) amplification of the *cytb* gene, as described in our previous study [14]. The complete *cytb* sequences (1140 bp) were aligned using CLUSTAL W and analyzed using the IQ-TREE web server [32].

### Rapid amplification of cDNA ends (RACE) PCR

RACE PCR was performed to acquire the 3′ and 5′ terminal genome sequences, using a SMARTer^®^ RACE 5′/3′ Kit (Takara Bio, Cat# 634858, Kusatsu, Japan) in accordance with the manufacturer’s guidelines. Each reaction used 2 µg of total RNA as input. Poly(A)tails were added with *Escherichia coli* poly(A) polymerase (New England Biolabs, Cat# M0276, Ipswich, MA, USA) following the manufacturer’s instructions. PCR products from both directions were sequenced using Sanger sequencing.

### Molecular screening of Paramyxoviruses from *Alexandromys fortis* samples

Total RNA was extracted from the kidney tissues of *A*. *fortis* using TRIzol™ Reagent (Thermo Fisher Scientific, Cat# 15596018, Waltham, MA, USA) according to the manufacturer’s protocol. cDNA was synthesized using the High-Capacity RNA-to-cDNA Kit with 1 µg of total RNA as input, following the kit instructions (Applied Biosystems, Cat# 4368814, Foster City, CA, USA). Paramyxovirus screening was performed using nested PCR targeting the RNA-dependent RNA polymerase region using primer sets specific for *Respirovirus*–*Morbillivirus*–*Henipavirus* (RMH), pan-paramyxovirus (pan-PAR), and *Avulavirus*–*Rubulavirus* (AVU-RUB), as previously described [33].

Initial and nested PCRs were conducted in 20-μL reaction volumes, each containing 0.05 µL of SuperTherm DNA polymerase at a stock concentration of 250 U/µL (JMR Holdings, Cat# JMR-801, Kent, UK), 1.5 μL of cDNA, and 10 μM of each primer. Thermal cycling conditions included an initial denaturation at 95 ℃ for 5 min, followed by six cycles at 94 ℃ for 40 s, annealing at 37 ℃ for 40 s, and elongation at 72 ℃ for 1 min; this was followed by 32 cycles of denaturation at 94 ℃ for 40 s, annealing at 42 ℃ for 40 s, and elongation at 72 ℃ for 1 min (SimpliAmp Thermal Cycler, Applied Biosystems, Cat# A24811, Carlsbad, CA, USA).

PCR products were purified using the MinElute^®^ PCR purification kit (Qiagen, Cat# 28,004, Hilden, Germany), and bidirectional Sanger sequencing was performed using the BigDye Terminator v3.1 Cycle Sequencing Kit (Applied Biosystems, Cat# 4337455, Waltham, MA, USA) on an automated sequencer (ABI 3730XL DNA Analyzer, Applied Biosystems, Cat# 3730XL, Waltham, MA, USA). The forward and reverse sequences were assembled and manually verified using SeqMan (DNASTAR Inc., Cat# LSMW-D, Madison, WI, USA). Consensus sequences were generated by inspecting electropherogram peaks to resolve mismatches, and bases were accepted only when supported by both forward and reverse reads. The sequence consensus was determined using nucleotide Basic Local Alignment Search Tool (BLASTn) from the National Center for Biotechnology Information (NCBI) RefSeq database [34].

### Metagenomic sequencing and de novo assembly

A total of 12 *A*. *fortis* specimens were selected for metagenomic next-generation sequencing to represent the diversity of PCR-positive detections obtained using different primer sets and to include samples suitable for exploratory viral detection based on RNA quality. For metagenomic library construction, 2.0–8.0 µg of total RNA extracted was used as input. Libraries were prepared using the TruSeq Stranded Total RNA Library Prep Gold Kit with Illumina Ribo-Zero Plus rRNA Depletion (Illumina, Cat# 20040529, San Diego, CA, USA), following the manufacturer’s protocol. High-throughput sequencing was performed by paired-end 100 sequencing on a NovaSeq platform (Illumina, Cat# 20012850, San Diego, CA, USA).

Sequencing quality was assessed prior to downstream analysis. All libraries met standard Illumina quality control criteria, with Q20 values ≥ 96.6% and Q30 values ≥ 91.4% in all samples. No quality-related issues were observed during read processing or assembly. Adapter sequences were trimmed, and host-derived sequences were removed using CLC Genomics Workbench v.24.0.1 (CLC Bio, Qiagen, Cat# 832021, Hilden, Germany). Host read subtraction was performed using the *Microtus fortis* reference genome (RefSeq accession: GCF_014885135.2). The remaining reads were quality filtered and assembled de novo in CLC. Assembled contigs were analyzed using BLASTn (v2.6.0) against the NCBI RefSeq viral genome database and further evaluated using the VirPipe pipeline, which performs agnostic taxonomic classification of nonhost contigs and enables detection of any viral sequences present in the dataset [34–36]. Contigs corresponding to paramyxoviruses and hepaciviruses were extracted and annotated to determine coding regions. Final genome reconstruction was achieved by aligning the raw reads to the validated de novo-assembled viral contigs to assess coverage and refine the consensus, using a minimum depth threshold of 10 × per base.

### Genomic characterization and prediction of signal peptidase cleavage sites

Viral genomic sequences were annotated using Geneious Prime (version 2023.2.1; Dotmatics, Cat# GP2023-2.1, Auckland, New Zealand) [37]. The open reading frames (ORFs) were translated and compared with known protein sequences using protein BLAST (BLASTp). The resulting genome was used as a reference for the phylogenetic analysis. CLUSTAL Omega 1.2.2 was used for the alignment of the translated ORF with the curated viral CDS, and the similarity percentage was determined using the BLOSUM62 matrix [38]. Predictions of putative cellular signal peptidase cleavage sites were made using artificial neural networks and hidden Markov models through the SignalP 6.0 Server and sequence homology [39].

### Analysis of N-linked glycosylation and zoonotic potential

The PyAPV glycoprotein sequence was analyzed for potential N-linked glycosylation sites using NetNGlyc-1.0 [40]. The zoonotic potential of the viral genomic sequences was assessed using a genome-based machine-learning model that evaluates host range associated genomic features [41]. A cutoff value of 0.293 was used, with predicted zoonotic potential categorized as follows: very high, indicated by the entire confidence interval (CI) exceeding the cutoff value; high, where the mean prediction surpassed the cutoff value and the CI also crossed it; medium, where the mean prediction was less than or equal to the cutoff and the CI crossed the cutoff; and low, determined by the entire 95% CI of the predicted probability below the cutoff value.

### Phylogenetic and cophylogenetic analysis

The genome sequences were aligned and trimmed using CLUSTAL Omega with Geneious Prime (version 2023.2.1; DotMatics, Cat# GP2023-2.1, Auckland, New Zealand). Whole-genome phylogenetic trees were generated using maximum likelihood methods according to the best-fit substitution model in the IQ-TREE web server with 1000 bootstrapping iterations. The generated tree was processed and graphically adjusted using the Interactive Tree of Life (iTOL) version 6 program [42]. Partial phylogenetic trees were generated using the maximum likelihood (ML) method with the best-fit evolutionary model LG + G + F with 1000 bootstrapping iterations in MEGA X. The event-based program eMPRess was employed to ascertain the comparative frequency of cross-species transmission and virus–host co-divergence (co-evolution) [43]. Maximum likelihood phylogenetic trees of the host mitochondrial cytochrome *b* and the complete genes of the viruses were used to reconcile the symbiont and host trees on the basis of the premise that a reduced cost enhances the likelihood of events in the duplication-transfer-loss model [44]. The costs for duplication, host-jumping (transfer), and extinction (loss) event types were set to 1.0, whereas the cost of virus–host co-divergence was considered a null event (*p* < 0.01).

## Results

### Epidemiology of novel paramyxovirus and hepacivirus of *Alexandromys fortis*

A total of 258 *A*. *fortis* specimens (97 males and 161 females) were collected from four rural areas in Gyeonggi Province (Paju, Pyeongtaek, Pocheon, and Yeoncheon) in 2001−2009. Specimens were categorized by year, trapping location, and sex. Most of the specimens were collected from Pyeongtaek (84.0%), whereas the rest of the small mammals were obtained from Paju (8.5%), Pocheon (5.0%), and Yeoncheon (2.0%). Paramyxovirus screening of *A*. *fortis* yielded 67 positive detections, corresponding to an overall prevalence of 26.0%. Positive results for *A*. *fortis* were higher in males (31.0%) than in females (21.9%) (Table 1). The highest rate of paramyxoviruses was detected in 59/258 (22.9%) *A*. *fortis* individuals using specific primers targeting *Respirovirus*, *Morbillivirus*, and *Henipavirus*. The remaining positive detections were obtained using the pan-PAR and AVU-RUB primer sets, each detecting four specimens. On the basis of PCR positivity and RNA quality, 12 representative specimens were selected for downstream metagenomic next-generation sequencing (mNGS): six RMH-positive specimens, two pan-PAR-positive specimens, and four AVU-RUB-positive specimens. Species identification of *A*. *fortis* (formerly *Microtus fortis*) was confirmed by sequencing of the mitochondrial DNA (mtDNA) cytochrome *b* (*cytb*) gene (Additional file 1).

**Table 1:** **The epidemiological characteristics of paramyxovirus screening in *Alexandromys fortis***

Year	District	Total samples	Total RNA positivity (%)^#^	RNA positivity by sex (%)^#^	
				Male	Female
2001	Paju	3	1/3 (33.3)	1/2 (50.0)	0/1
	Yeoncheon	1	0/1	0/1	0/0
2003	Paju	10	2/10 (20.0)	1/5 (20.0)	1/5 (20.0)
2004	Paju	7	1/7 (14.3)	0/1	1/6 (16.7)
	Pocheon	12	2/12 (16.7)	0/2	2/10 (20.0)
	Yeoncheon	2	0/2	0/0	0/2
2005	Paju	2	2/2 (100)	0/0	2/2 (100)
	Pocheon	1	0/1	0/0	0/1
	Yeoncheon	3	2/3 (66.7)	1/1 (100)	1/2 (50.0)
2006	Pyeongtaek	5	3/5 (60.0)	1/1 (100)	2/4 (50.0)
2009	Pyeongtaek	212	54/212 (25.5)	26/84 (31.0)	28/128 (21.9)
Total		258	67/258 (26.0)	30/97 (30.9)	37/161 (23.0)

^#^RNA positivity was defined by reverse transcription PCR (RT-PCR) detection of a partial L gene fragment using primers targeting *Orthoparamyxovirinae*. Viral lineage assignment was performed during downstream metagenomic analyses.

### Metagenomic next-generation sequencing of PyAPV

mNGS of the six RMH-positive specimens yielded 17 291 696−127 359 968 paired-end reads per sample (Af01-01, Af04-04, Af05-01, Af05-12, Af09-05, and Af09-122). Total reads of each sample were used for de novo assembly, generating up to 2300 contigs with lengths ranging from 1 to 20 kb. The identified contigs matching members of the genus *Jeilongvirus* revealed percentage identities of less than 85.0% with the ORFs corresponding to the respective CDSs. Consensus validation of identified virus, Pyeongtaek Alexandromys paramyxovirus (PyAPV), was achieved by remapping the matched sequences with raw reads.

**Figure 1:**
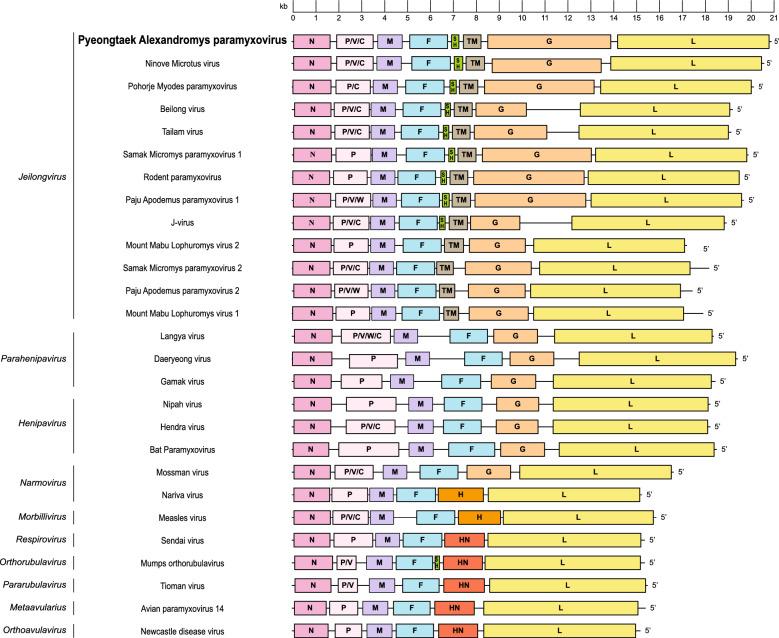
**Genome organization of Pyeongtaek Alexandromys paramyxovirus (PyAPV) with representative paramyxoviruses within the family *Paramyxoviridae***. The genome organization of PyAPV is shown in comparison with the genus *Jeilongvirus* and other genera within the family *Paramyxoviridae*. Black lines adjacent to the *N* and *L* genes symbolize the untranslated regions (UTRs). Abbreviations: N, nucleocapsid protein; P, phosphoprotein; M, matrix protein; F, fusion protein; SH, small hydrophobic protein; TM, transmembrane protein; G, glycoprotein; H, hemagglutinin protein; L, large protein.

**Figure 2:**
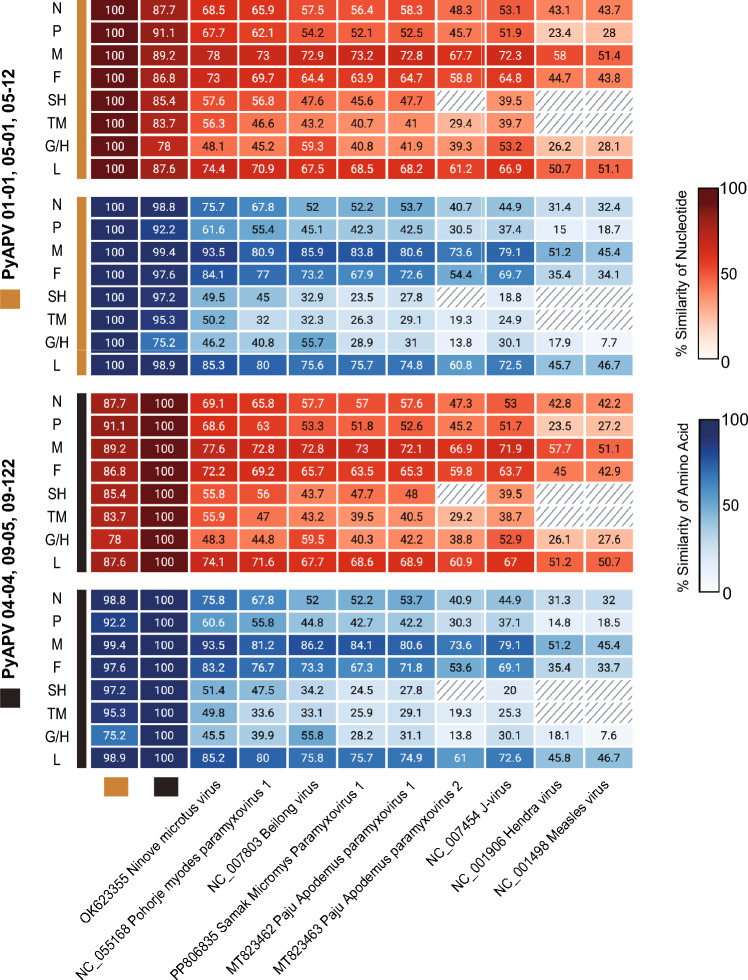
**Nucleotide and amino acid similarity between Pyeongtaek Alexandromys paramyxovirus (PyAPV) and other members of the family *Paramyxoviridae***. A heatmap illustrates the similarity percentages of nucleotides and amino acids for each gene, where red represents nucleotide similarity and blue indicates amino acid similarity. The shaded region in the heatmap indicates where the respective gene is not present in the species. Abbreviations: N, nucleocapsid protein; P, phosphoprotein; M, matrix protein; F, fusion protein; SH, small hydrophobic protein; TM, transmembrane protein; G, glycoprotein; H, hemagglutinin protein; HN, hemagglutinin-neuraminidase protein; L, large protein.

All PyAPV genomes had near-complete genomes of 20 874 nt. The geographic distribution was observed in Paju 3/6 (50.0%), Pyeongtaek 2/6 (33.3%), and Yeoncheon 1/6 (16.7%) (Additional file 2). The nearly complete PyAPV genomes constructed from samples Af01-1, Af05-1, and Af05-12 exhibited distinct characteristics compared with those from Af04-4, Af09-5, and Af09-122 (Additional file 3). GC content varied slightly between the two groups: 40.4–40.5% in Af01-1, Af05-1, and Af05-12; and 39.8–39.9% in Af04-4, Af09-5, and Af09-122. Amplification of the 3′ termini of PyAPV by RACE PCR was unsuccessful, while 5′ RACE extended the genome by 29 nucleotides. The genome organization of PyAPV consists of eight genes arranged in the order 3′–N–P/V/C–M–F–SH–TM–G–L–5′ (Figure 1), with the most notable differences observed in the *G* gene (Additional file 4). The start, stop, and intergenic region sequences of PyAPV are summarized in Table 2. Comparative analyses of gene organization and amino acid sequences between PyAPV and other paramyxoviruses are presented in Figure 2 and Additional files 5 and 6. However, the contig of hantaviruses, arenaviruses, or other rodent-associated viral families was undetectable in this study.

**Table 2:** **Sequence of intergenic regions (IGR) and transcriptional start and stop signals of Pyeongtaek Alexandromys paramyxovirus (PyAPV)**

	Genes	Gene stop	IGR	Gene Start
PyAPV 01-01, 05-01, 05-12	/N	–	CTT	ATGTCTTCTC
	N/P	TAACTCTTAA	CTT	ATGGCAGTAT
	P/M	GGACCAATGA	CTT	ATGGCAGGCA
	M/F	CATGAAGTGA	CTT	ATGGTGTCAA
	F/SH	TGTTGGGTGA	CTT	ATGAATCCTA
	SH/TM	CAGAGACTAA	CTT	ATGGTCGCAG
	TM/G	CATGAAATAG	CTT	ATGAATCAGT
	G/L	GTATAAGTAA	CTT	ATGGCTACTC
	L/	GGATGAGTAA	–	–
PyAPV 04-04, 09-05, 09-122	/N	–	CTT	ATGTCTTCTC
	N/P	AAATTCGTAA	CTT	ATGGCAGTGT
	P/M	TCAACAGTGA	CTT	ATGGCGGGCA
	M/F	CATGAAGTGA	CTT	ATGAAAGCAA
	F/SH	TGTTGGATGA	CTT	ATGAATCCTA
	SH/TM	CAGAGACTAG	CTT	ATGGTCGCAG
	TM/G	CATGAAATAG	CTT	ATGAATCAGT
	G/L	GTACAAGTAA	CTT	ATGGCTACCC
	L/	GGACGAGTAA	–	–

*PyAPV*, Pyeongtaek Alexandromys paramyxovirus; *N*, nucleocapsid protein; *P*, phosphoprotein; *M*, matrix protein; *F*, fusion protein; *SH*, small hydrophobic protein; *TM*, transmembrane protein; *G*, glycoprotein; *H*, hemagglutinin protein; *HN*, hemagglutinin-neuraminidase protein; *L*, large protein.

### Metagenomic next-generation sequencing and genomic organization of Hepacivirus J–Alexandromys

mNGS of the four AVU-RUB-positive specimens (Af09-31, Af09-84, Af09-195, and Af09-207) generated between 72 036 796 and 111 130 158 paired-end reads per sample. The de novo assembly resulted in 103–2303 contigs with lengths ranging from 1 to 9 kb. All four samples yielded contigs corresponding to a rodent-associated hepacivirus, and paramyxovirus-related contigs were undetected.

The near-complete genome of Hepacivirus J–Alexandromys is 9567 nucleotides in length with a GC content of 54.0%. Attempts to extend the terminal regions of genome using RACE PCR were unsuccessful. Hepacivirus J–Alexandromys was found exclusively in Pyeongtaek 4/4 (100%) of the *A*. *fortis* specimens.

**Figure 3:**
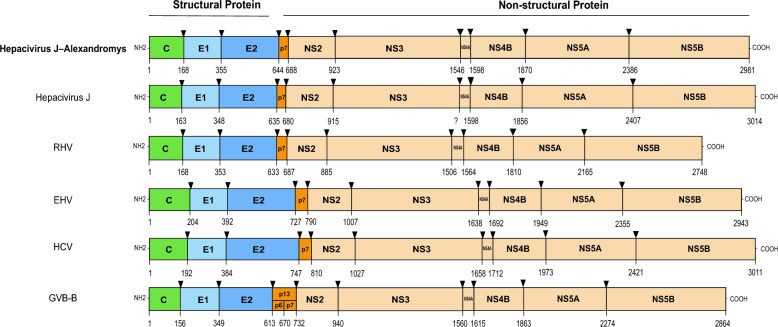
**Predicted cleavage sites of Hepacivirus J–Alexandromys**. Predictions of the putative cellular signal peptidase cleavage sites are shown in comparison with Hepacivirus J, rodent hepacivirus (RHV), equine hepacivirus (EqHV), hepatitis C virus (HCV), and GB virus-B (GBV-B). Scaled boxes represent the respective mature proteins. Abbreviations: C, core protein; E1, envelope glycoprotein 1; E2, envelope glycoprotein 2; NS, nonstructural protein.

**Figure 4:**
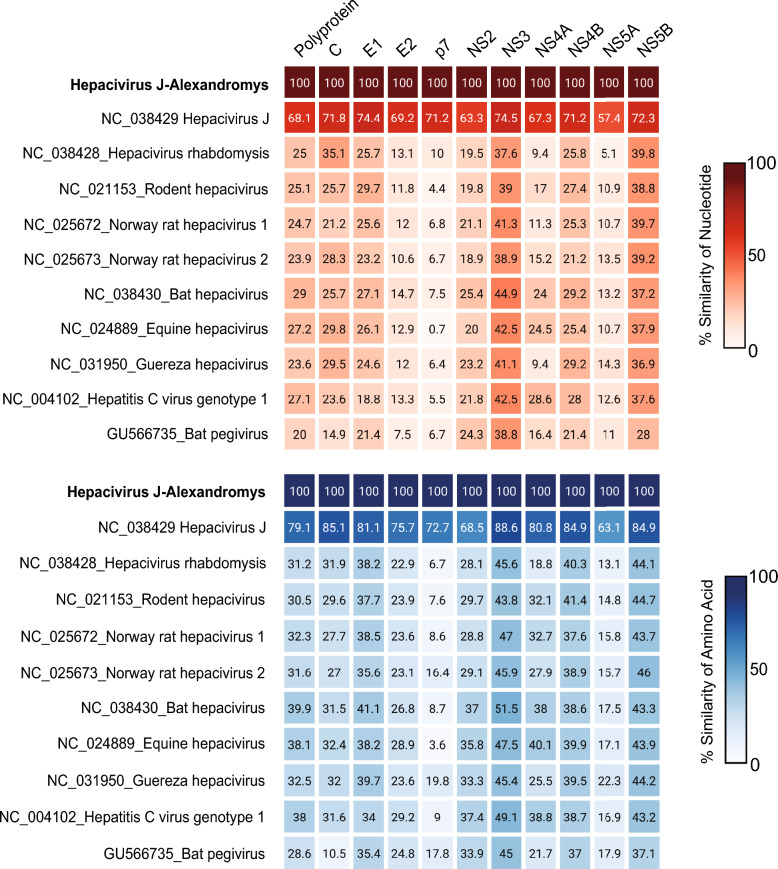
**Nucleotide and amino acid similarity of Hepacivirus J–Alexandromys and representative members of the family *Flaviviridae***. The heatmap shows similarity percentages of the polyprotein coding sequence, where red indicates nucleotide similarity and blue indicates amino acid similarity. Color scale is presented with the representative gradient.

The genome consists of a single 5′–Polyprotein–3′ open reading frame, typical of the *Hepacivirus* genus. The predicted cleavage sites on the polyprotein yielded 10 viral proteins characteristic of hepaciviruses: three structural proteins (core, E1, and E2) and seven nonstructural proteins (p7, NS2, NS3, NS4A, NS4B, NS5A, and NS5B) (Figure 3). The virus shared 79.1% of amino acid similarity with *Hepacivirus glareoli*, a representative member of the species *Hepacivirus J* (Figure 4, Additional files 7 and 8). Annotated genome sequences of PyAPV and Hepacivirus J–Alexandromys have been deposited in GenBank, with accession numbers provided in Additional file 9.

### Phylogenetic and cophylogenetic analysis of novel PyAPV and Hepacivirus J–Alexandromys

**Figure 5:**
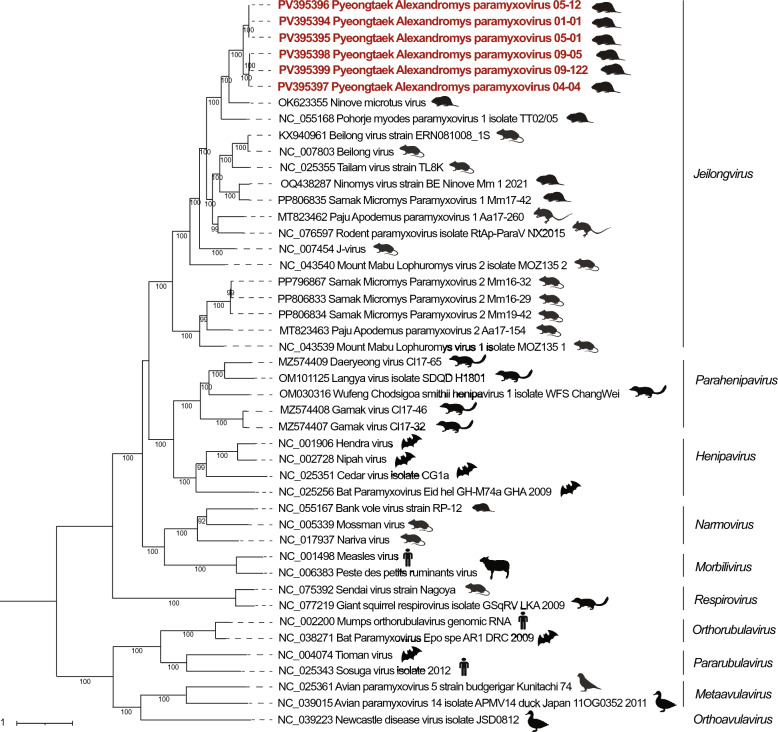
**Phylogenetic tree of Pyeongtaek Alexandromys paramyxovirus (PyAPV) within the family *Paramyxoviridae***. The phylogenetic tree was generated using maximum likelihood analysis by IQTREE web server, with GTR + F + I + G4 model chosen according to BIC and 1000 bootstrapping. PyAPV samples from this study are shown in red. GenBank accession numbers of reference viruses are shown on the right of taxon names. The scale bar indicates genetic distance, while the icon adjacent to the virus name signifies its common host.

Nearly whole-genome sequences of the PyAPV formed two consistent genetic clusters, both of which shared a common ancestor with Ninove Microtus virus (species *Jeilongvirus microti*) and *Pohorje Myodes paramyxovirus 1* isolate TT02/05 (Figure 5). These clusters, represented by sequences from Af01-1, Af05-1, and Af05-12, as well as Af04-4, Af09-5, and Af09-122, were consistently formed in all gene phylogenies. Phylogenetic analysis based on complete large (L) protein amino acid sequences showed branch lengths of 0.21–0.22 from Ninove Microtus virus to each cluster, respectively (Additional file 10). However, the branch length between the two PyAPV clusters was less than 0.03, which does not meet the threshold for species demarcation within the genus *Jeilongvirus*. These results support the classification of PyAPV as a single novel species and genetically distinct from known *Jeilongvirus* members [12].

**Figure 6:**
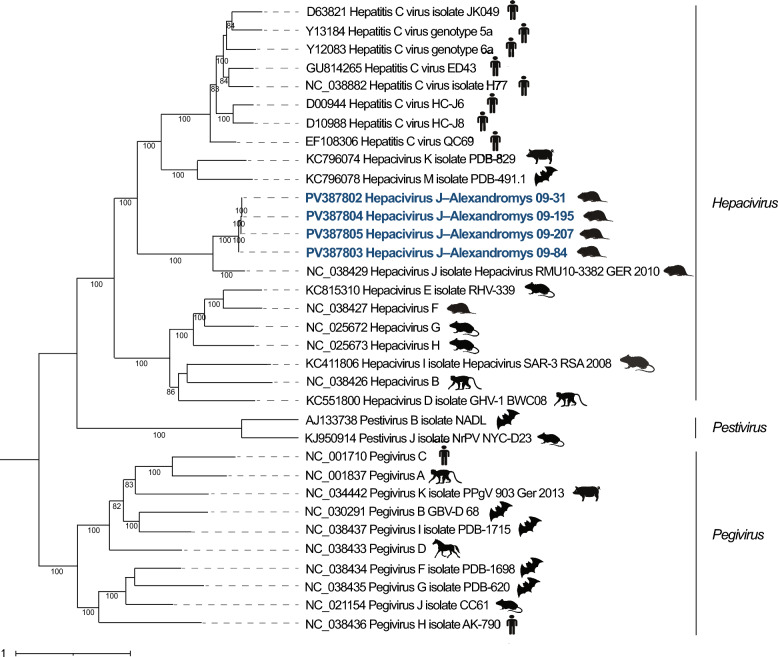
**Phylogenetic tree of Hepacivirus J–Alexandromys within the family *Flaviviridae***. The phylogenetic tree was generated using maximum likelihood analysis with GTR + F + I + G4 model chosen according to BIC and 1000 bootstrapping by IQTREE web server. GenBank accession numbers of reference hepaciviruses are listed to the right of the taxon names. The scale bar symbolizes the genetic distance of each hepacivirus reference. Host symbols indicate the source host species for each virus within the genera *Hepacivirus*, *Pegivirus*, and *Pestivirus.*

The lineage containing Hepacivirus J–Alexandromys and *Hepacivirus glareoli* formed a distinct clade separate from other hepaciviruses known to infect humans (*Hepacivirus C*), pigs (*Hepacivirus K*), and bats (*Hepacivirus M*) (Figure 6). Pairwise p-distance comparisons between Hepacivirus J–Alexandromys and *Hepacivirus glareoli* yielded values of 0.106 for NS3 and 0.148 for NS5B, both below the ICTV species demarcation thresholds, supporting classification within the species *Hepacivirus J* (Additional file 11).

**Figure 7:**
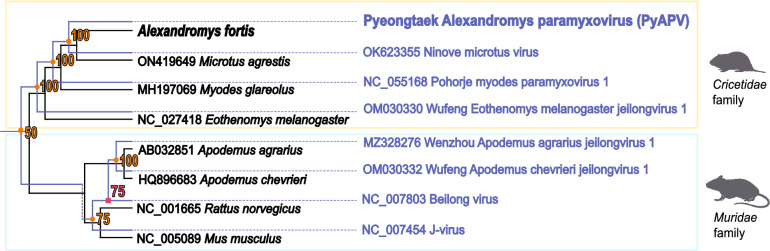
**Cophylogenetic analysis of Pyeongtaek Alexandromys paramyxovirus (PyAPV) with the reservoir hosts**. Cophylogenetic analysis of Pyeongtaek Alexandromys paramyxovirus with members of *Jeilongvirus* and the corresponding hosts. The black phylogenetic tree represents the host tree, derived from mtDNA *cytb* sequences, while the blue phylogenetic tree represents the viral tree. The likelihood of occurrence is expressed as a percentage (%) and is accompanied by different symbols: orange circles for co-divergence, pink squares for host-switch (transfer) events, purple diamonds for duplication events, and dashes for gene loss. Blue arrows indicate the directionality of transfer events.

Cophylogenetic analysis revealed six-divergence events, one host-jumping (transfer) event, and one extinction (loss) event. PyAPV clustered closely with other Jeilongviruses associated with *Cricetidae* rodents and forming a lineage distinct from *Muridae*-associated viruses. This congruent topology supports host–virus co-divergence and family-level host specificity in *Jeilongvirus* evolution, with no evidence of recent host-switching events (Figure 7). The inferred evolutionary history suggests that PyAPV and *Ninove Microtus virus* share a common ancestral lineage associated with *Microtus* voles, followed by parallel diversification in their respective hosts.

### Analysis of N-linked glycosylation (NLG)

We identified potential NLG sites in the whole amino acid sequences of PyAPV G proteins (Additional file 12). Nine putative NLG sites were predicted at positions 56, 423, 508, 515, 694, 908, 1000, 1150, and 1453 in strains PyAPV 01-01, 05-01, and 05-12, all exceeding the threshold value of 0.6. By contrast, strains PyAPV 04-04, 09-05, and 09-122 harbored eleven predicted NLG sites at positions 56, 423, 508, 515, 694, 831, 977, 1154, 1326, 1453, and 1558. The overall glycosylation patterns of PyAPV were broadly comparable to those of Ninove Microtus virus and Pohorje Myodes paramyxovirus 1, suggesting conserved structural features within this lineage.

### Zoonotic potential of novel PyAPV and Hepacivirus J–Alexandromys

**Figure 8:**
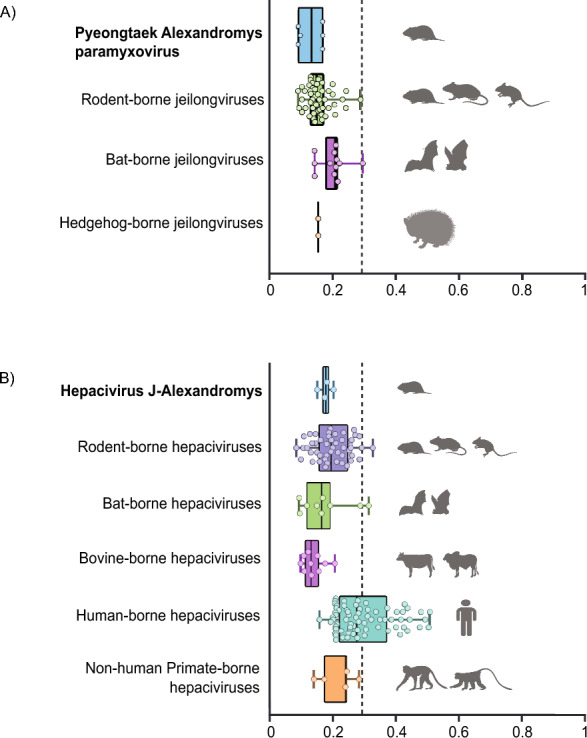
**Zoonotic potential of Pyeongtaek Alexandromys paramyxovirus (PyAPV) and Hepacivirus J–Alexandromys**. **A** Zoonotic potential of Pyeongtaek Alexandromys paramyxovirus (PyAPV) compared with other *Jeilongvirus* members found in different hosts. **B** Zoonotic potential of Hepacivirus J–Alexandromys compared with other *Hepacivirus* members in their respective hosts. The circles represent mean prediction score, while boxplots represent the distribution of area under the receiver operating characteristic (AUROC) scores for viruses within each host group. Host symbols indicate the source host species for each virus.

The zoonotic potential of PyAPV and Hepacivirus J–Alexandromys was assessed using a genome-based machine learning model. PyAPV was classified as having medium zoonotic potential, with a mean prediction score of 0.187 falling just below the model’s threshold. However, the associated confidence interval (CI 0.095–0.357) crossed the threshold, suggesting a borderline classification (Figure 8A). Similarly, Hepacivirus J–Alexandromys was also predicted to have medium zoonotic risk (Figure 8B). These findings are consistent with prior assessments of rodent-associated *Jeilongvirus* and *Hepacivirus* species, many of which are categorized as having intermediate potential for zoonotic spillover.

## Discussion

In this study, we found one novel paramyxovirus, Pyeongtaek Alexandromys paramyxovirus (PyAPV), and one rodent-associated hepacivirus classified within the species *Hepacivirus J* from *Alexandromys fortis* specimens collected in the ROK. A total of 10 near-complete genomes were obtained, including 6 genomes for PyAPV and 4 genomes for the *A*. *fortis* hepacivirus. The PyAPV genomes formed two consistent genetic clusters despite originating from the same host species. Among PyAPV genes, the *G* gene showed the lowest amino acid identity between clusters (75.2%), suggesting substantial divergence. The variability of the *G* gene are consistent with features reported for other Jeilongviruses where extended and heterogeneous G proteins are commonly observed [11]. Sequence variation in the attachment glycoprotein of *Paramyxoviridae* has been associated with immune-driven selection and receptor interactions in respiratory syncytial virus and henipaviruses [45, 46]. In this context, similar selective pressures might contribute to the observed variation in the PyAPV *G* gene. Predicted N-linked glycosylation sites were largely conserved between the two PyAPV clusters, with only limited positional differences. N-linked glycosylation has been shown to influence glycoprotein folding, stability, and receptor interactions in diverse viruses [47]. The conservation of glycosylation patterns indicates that PyAPV G proteins retain similar structural and functional constraints despite sequence variation. Further studies, including site-specific selection analyses and functional characterization, might provide biological significance of this variation.

PyAPV clustered within the genus *Jeilongvirus*, showing the highest sequence similarity to Ninove Microtus virus previously reported in *Microtus agrestis* from Belgium [48]. Despite this relationship, pairwise nucleotide identities remained low (62.2–62.4%). Cophylogenetic analyses revealed congruent host–virus evolutionary histories between *A*. *fortis*–PyAPV and *M*. *agrestis*–Ninove Microtus virus, consistent with long-term host associations. Moreover, phylogenetic branch lengths of L protein amino acid sequences exceeded the ICTV species demarcation threshold (0.03), supporting PyAPV as a novel paramyxovirus in *A*. *fortis*.

Hepacivirus J–Alexandromys formed a monophyletic lineage with *Hepacivirus glareoli*, a representative virus of the species *Hepacivirus J*. Pairwise p-distance values for NS3 (0.106) and NS5B (0.148) were below ICTV species demarcation thresholds, supporting classification within the species *Hepacivirus J* rather than a novel species. The clustering of hepaciviruses detected in *Alexandromys* and *Myodes* hosts suggests host-associated lineages within this species. Although the present dataset is limited to rodent-associated viruses, previous large-scale studies have shown that early-diverging hepacivirus lineages in bats and rodents, consistent with long-term circulation in small mammal hosts and historical cross-species transmission events [19, 49]. These findings highlight the diversity of rodent hepaciviruses and demonstrate the importance of broader sampling to improve resolution of variation within the genus *Hepacivirus*.

Cophylogenetic analysis revealed congruent host–virus evolutionary patterns between PyAPV from *A*. *fortis* and *Microtus*-associated jeilongviruses. The phylogenetic proximity of PyAPV and *A*. *fortis* hepacivirus to viruses from European rodents may be attributed to the intricate evolutionary and biogeographic history of Eurasian microtines. Fossil and genomic evidence demonstrate at least three independent colonization events of *Microtus* voles from Asia into Europe and North America [50]. These dispersal events, combined with relatively recent diversification of Eurasian *Microtus* lineages, provide a plausible context for the emergence of related viral lineages in rodents occupying similar ecological niches in Europe and East Asia [51, 52]. The cophylogenetic signal observed for PyAPV, which was dominated by codivergence events, is consistent with this broader pattern of long-term host–virus association described for other Jeilongviruses, including those characterized in bat and rodent hosts [53]. This host–virus co-distribution highlights the importance of considering rodent evolutionary history when evaluating the emergence of novel viruses relevant to veterinary health. Additional studies are needed to clarify whether these phylogenetic patterns reflect ancient host–virus associations shaped by historical biogeographic movements among rodent populations in Eurasia.

Genome-based machine learning models predicted medium zoonotic potential for both PyAPV and *A*. *fortis* hepacivirus. These findings are consistent with previous observations for other rodent-associated *Jeilongvirus* and *Hepacivirus* species [54]. The current assessments rely on in silico predictions and do not incorporate ecological context or experimental validation relevant to spillover risk. Therefore, these results warrant further investigation through biological validation, including experimental infections, receptor-binding assays, and serological studies in both animal and human populations.

This study has several limitations. RACE PCR did not recover complete terminal sequences for PyAPV and Hepacivirus J–Alexandromys, resulting in incomplete characterization of genomic termini. The lack of viral isolation restricts evaluation of replication kinetics, tissue tropism, and pathogenicity. All paramyxoviruses were confined to kidney tissues, consistent with the broad tropism reported for paramyxoviruses [55]. Hepaciviruses, on the other hand, are known to exhibit hepatotropism [19]. Notably, substantial replication in the liver can increase the likelihood of viral detection in other well-perfused tissues, such as the kidney, potentially explaining their detection site. Another limitation is the restricted geographic sampling of *A*. *fortis* populations within the ROK. Expanding ecological surveillance, host behavioral studies, and serological testing of sympatric domestic animals would improve understanding of transmission dynamics and potential host range.

In conclusion, our study demonstrates one novel *Jeilongvirus* species and a distinct *Hepacivirus J* lineage circulating in *A*. *fortis* in the ROK. These findings contribute to the expanding evidence that rodents serve as reservoirs for genetically diverse RNA viruses with zoonotic relevance. The concurrent discovery of two distinct RNA virus lineages, *Jeilongvirus* and *Hepacivirus*, in a single rodent species highlights the ecological complexity of virus–host associations and highlights the value of integrating metagenomics with evolutionary analyses to better understand virus emergence and host specificity. Overall, this study provides new insight into the genomic diversity, evolutionary relationships, and zoonotic risks of rodent-borne RNA viruses, aiding in the preparedness and response to emerging zoonotic diseases.

## Supplementary Information

**Additional file 1. Mitochondrial** ***cytb*** **phylogenetic tree of** ***Alexandromys fortis*** **in this study.** The phylogenetic tree was constructed using maximum likelihood analysis by IQTREE web server, with the TPM3u+F+G4 model chosen according to BIC and 1000 bootstrapping. The blue colored label is the mtDNA *cytb* sequences of the *Alexandromys fortis* in this study.

**Additional file 2. Distribution of paramyxovirus and hepacivirus positive samples in Gyeonggi province, ROK. **The map shows the trapping sites in Yeoncheon, Pocheon, Paju, and Pyeongtaek, four cities located in Gyeonggi province. Pie charts indicate the proportion of PCR-screening–positive samples for paramyxoviruses (red), hepacivirus-positive samples (orange), and PCR-screening–negative samples (blue) at each site. Colored circles mark sampling locations where viral genomes were confirmed by mNGS, with red indicating PyAPV-confirmed sites and orange indicating Hepacivirus J–Alexandromys–confirmed sites. The map was generated using QGIS version 3.38.0-Grenoble on a Windows 11 operating system.

**Additional file 3. Phylogenetic tree of Pyeongtaek Alexandromys paramyxovirus (PyAPV) partial genomes with other paramyxoviruses.** The phylogenetic tree was constructed using maximum likelihood analysis by IQTREE web server, employing the TPM3u+F+G4 model selected on the basis of BIC and incorporating 1000 bootstrapping iterations. The partial sequences generated two separate clusters, aligning with the phylogenetic tree derived from the complete genome sequences. N, nucleocapsid protein (A); P, phosphoprotein (B); M, matrix protein (C); F, fusion protein (D); SH, small hydrophobic protein (E); TM, transmembrane protein (F); G, glycoprotein; H, hemagglutinin protein; HN, hemagglutinin-neuraminidase protein (G); L, large protein (H).

**Additional file 4. Nucleotide and amino acid similarities between Pyeongtaek Alexandromys paramyxovirus (PyAPV) phylogenetic clusters.** The similarity between PyAPV clusters presented in percentage.

**Additional file 5. The nucleotide similarity of Pyeongtaek Alexandromys paramyxovirus (PyAPV) clusters in comparison to other** ***Paramyxoviridae* species.** The similarity is in percentage (%). N, nucleocapsid protein; P, phosphoprotein; M, matrix protein; F, fusion protein; SH, small hydrophobic protein; TM, transmembrane protein; G, glycoprotein; H, hemagglutinin protein; HN, hemagglutinin-neuraminidase protein; L, large protein. *H is the attachment protein in Measles virus; **HN is the attachment protein in Mumps orthorubulavirus, Tioman virus, Avian paramyxovirus 5, and Newcastle disease virus.

**Additional file 6. The amino acid similarity of Pyeongtaek Alexandromys paramyxovirus (PyAPV) clusters in comparison to other** ***Paramyxoviridae* species.** The similarity is in percentage (%). N, nucleocapsid protein; P, phosphoprotein; M, matrix protein; F, fusion protein; SH, small hydrophobic protein; TM, transmembrane protein; G, glycoprotein; H, hemagglutinin protein; HN, hemagglutinin-neuraminidase protein; L, large protein. *H is the attachment protein in Measles virus; **HN is the attachment protein in Mumps orthorubulavirus, Tioman virus, Avian paramyxovirus 5, and Newcastle disease virus.

**Additional file 7. The nucleotide similarity of Hepacivirus J–Alexandromys in comparison to other** ***Flaviviridae* species.** The nucleotide similarity between Hepacivirus J–Alexandromys with other *Flaviviridae* species.

**Additional file 8. The amino acid similarity of Hepacivirus J–Alexandromys in comparison to other** ***Flaviviridae* species.** The amino acid similarity between Hepacivirus J–Alexandromys with other *Flaviviridae* species.

**Additional file 9. List of GenBank accession number for viral and host sequences found in this study. **List of the GenBank submissions.

**Additional file 10. L Protein Phylogeny and Species Demarcation of Pyeongtaek Alexandromys paramyxovirus (PyAPV).** The phylogenetic tree was constructed on the basis of amino acid sequences of the L protein, using the alignment template provided by the ICTV for species demarcation within the *Paramyxoviridae* family. Branch lengths are indicated on each branch. PyAPV sequences identified in this study are highlighted in red. GenBank accession numbers of reference viruses are shown on the right of taxon names.

**Additional file 11. Pairwise patristic distance (PDD) of Hepacivirus J–Alexandromys.** Hepatitis C virus (HCV), GB virus-B (GBV-B), equine hepacivirus (EqHV).

**Additional file 12. The predicted N-glycosylation site of PyAPV glycoproteins in comparison with representative** ***Paramyxoviridae* members.** Multiple sequence alignment of full-length glycoproteins from ten *Jeilongvirus* members and one *Henipavirus* reference. N-linked glycosylation sites predicted with 9/9 consensus (“+” calls only) are indicated by vertical markers within pink bars representing glycoprotein length.

## Data Availability

All the viral and cytb sequences can be accessed at GenBank with accession number listed in Additional file 9.
