# Supplementary figures and images for: Molecular prevalence, genomic characterization, and zoonotic potential of novel paramyxovirus and hepacivirus in Alexandromys fortis, Republic of Korea
Source: Vet Res. 2026 May 28;57:96. doi: 10.1186/s13567-026-01777-z (PMC13220433; doi:10.1186/s13567-026-01777-z)

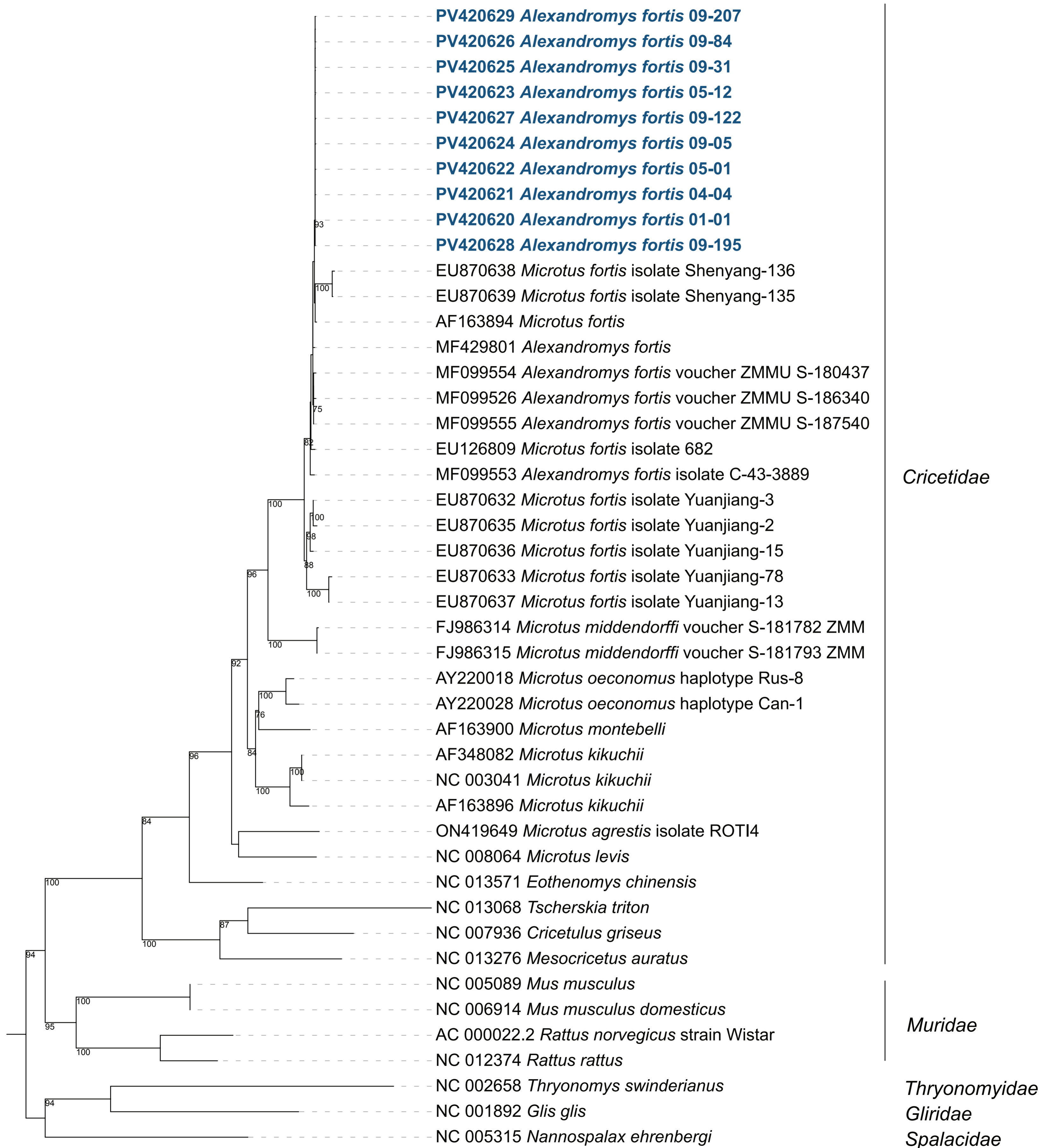

Supplement: Supplementary file 1 — Additional file 1. Mitochondrial cytb phylogenetic tree of Alexandromys fortis in this study. The phylogenetic tree was constructed using maximum likelihood analysis by IQTREE web server, with the TPM3u+F+G4 model chosen according to BIC and 1000 bootstrapping. The blue colored label is the mtDNA cytb sequences of the Alexandromys fortis in this study. [file 13567_2026_1777_MOESM1_ESM.pdf]

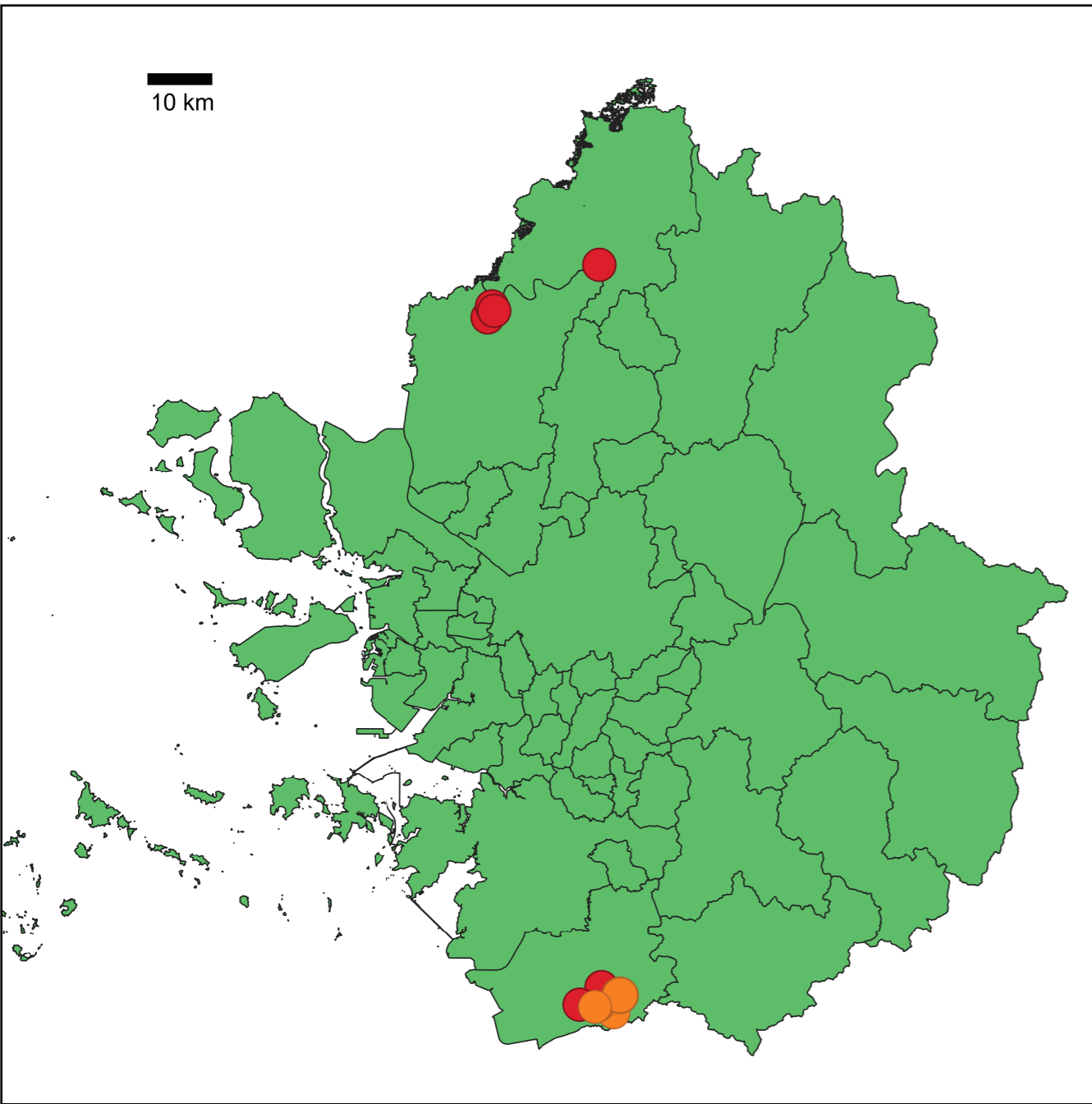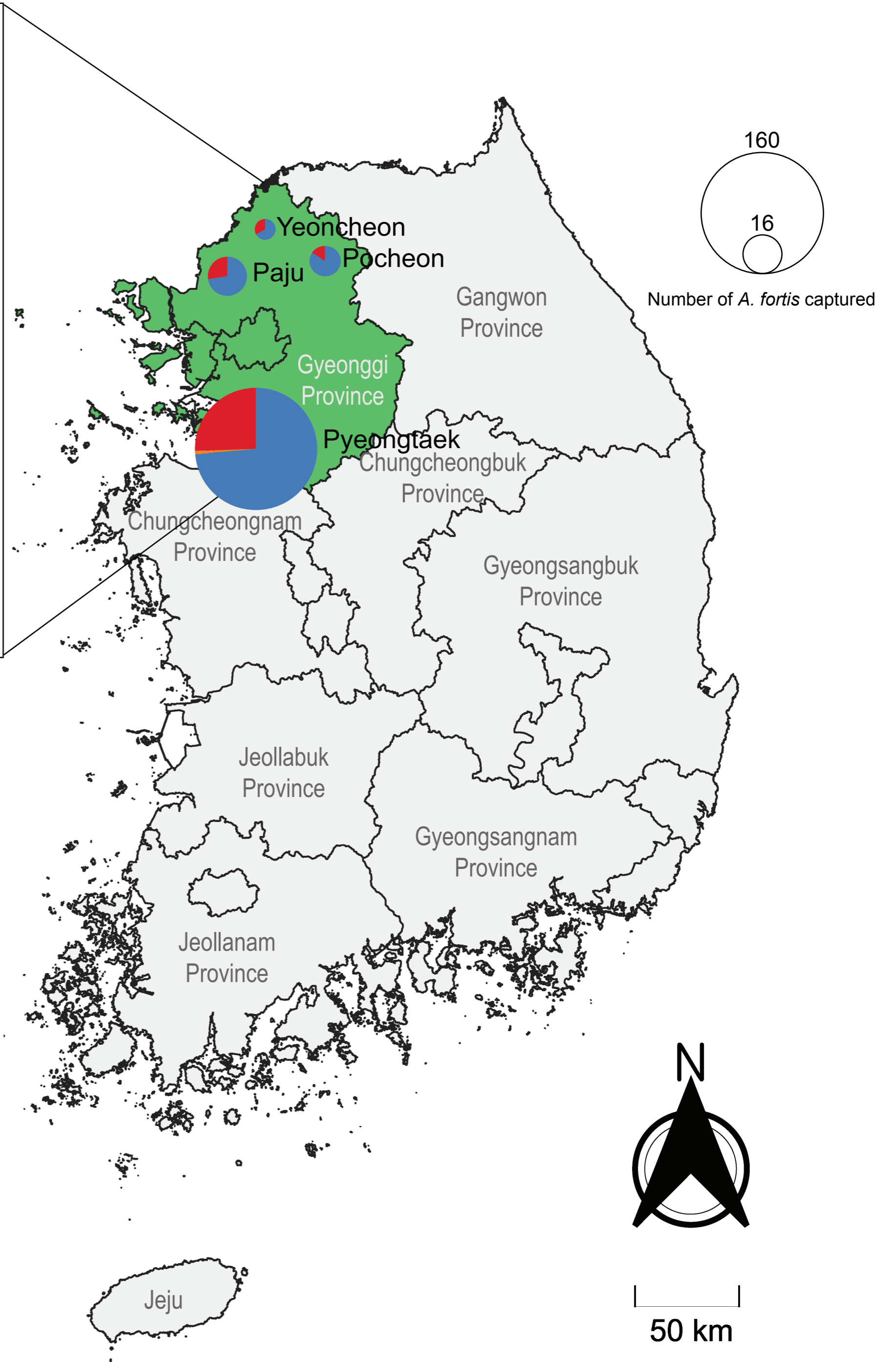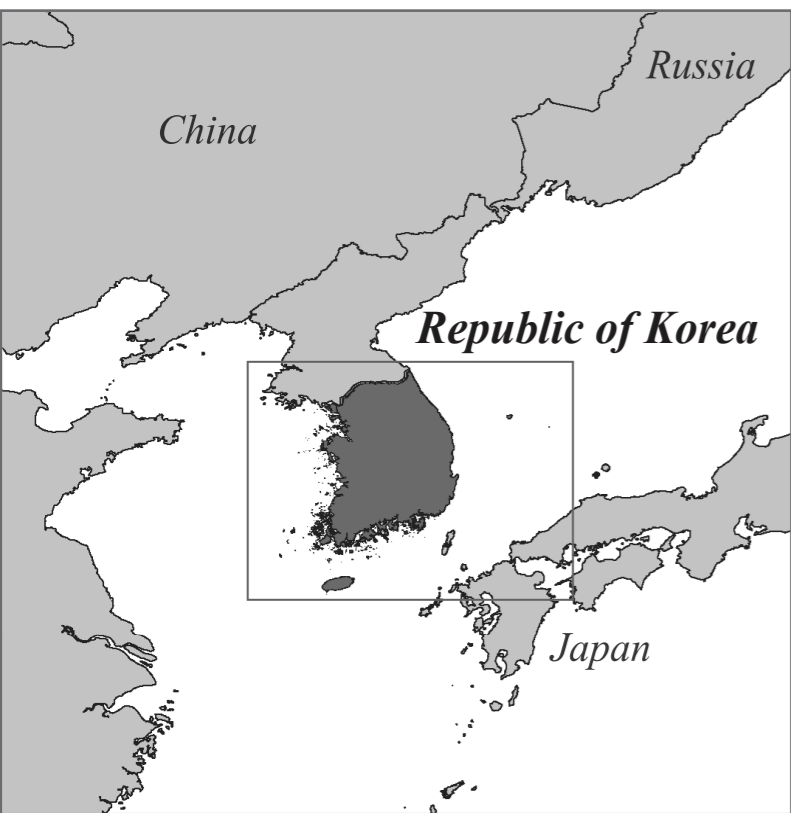

Supplement: Supplementary file 2 — Additional file 2. Distribution of paramyxovirus and hepacivirus positive samples in Gyeonggi province, ROK. The map shows the trapping sites in Yeoncheon, Pocheon, Paju, and Pyeongtaek, four cities located in Gyeonggi province. Pie charts indicate the proportion of PCR-screening–positive samples for paramyxoviruses (red), hepacivirus-positive samples (orange), and PCR-screening–negative samples (blue) at each site. Colored circles mark sampling locations where viral genomes were confirmed by mNGS, with red indicating PyAPV-confirmed sites and orange indicating Hepacivirus J–Alexandromys–confirmed sites. The map was generated using QGIS version 3.38.0-Grenoble on a Windows 11 operating system. [file 13567_2026_1777_MOESM2_ESM.pdf]

A)

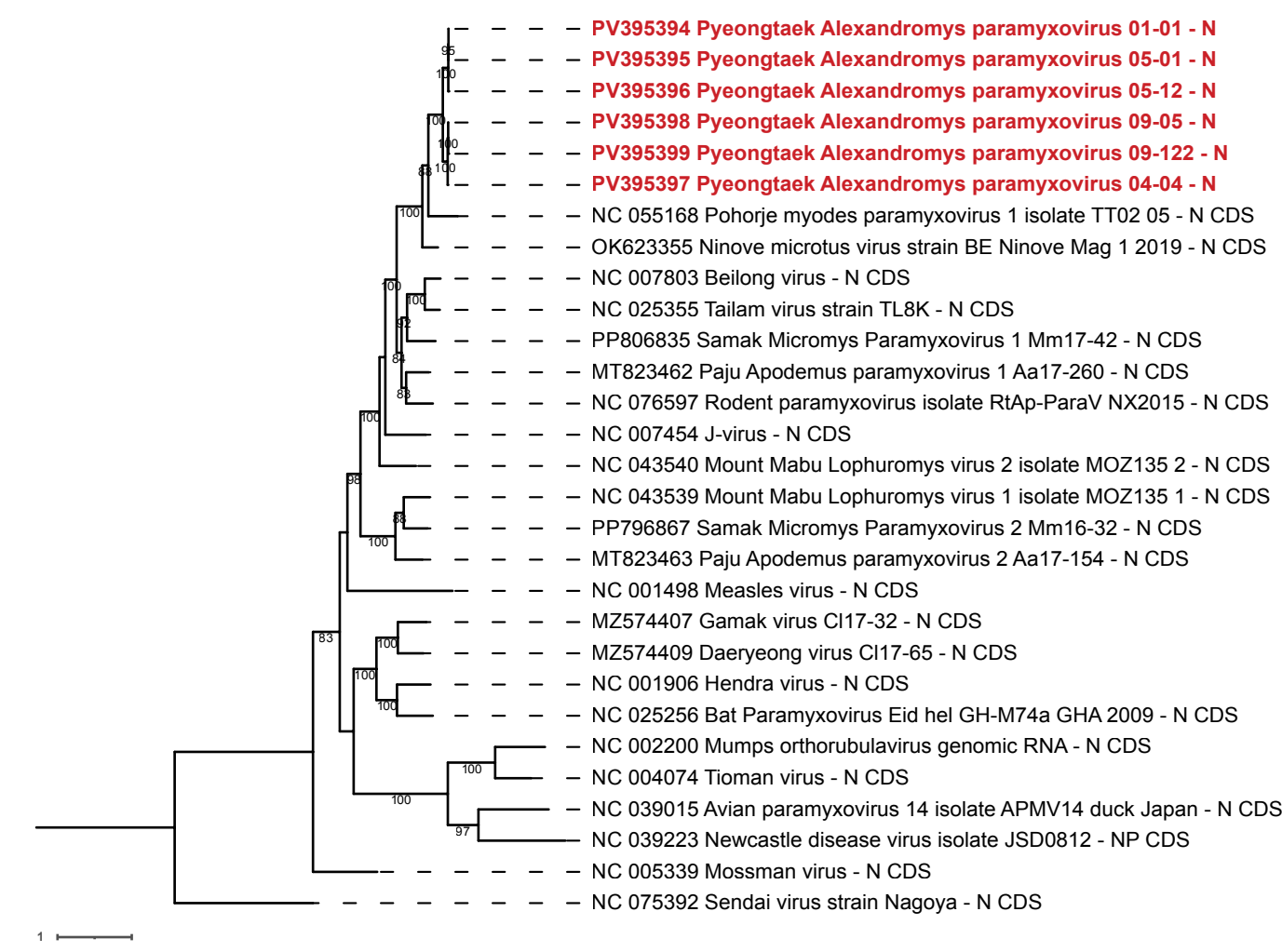

B)

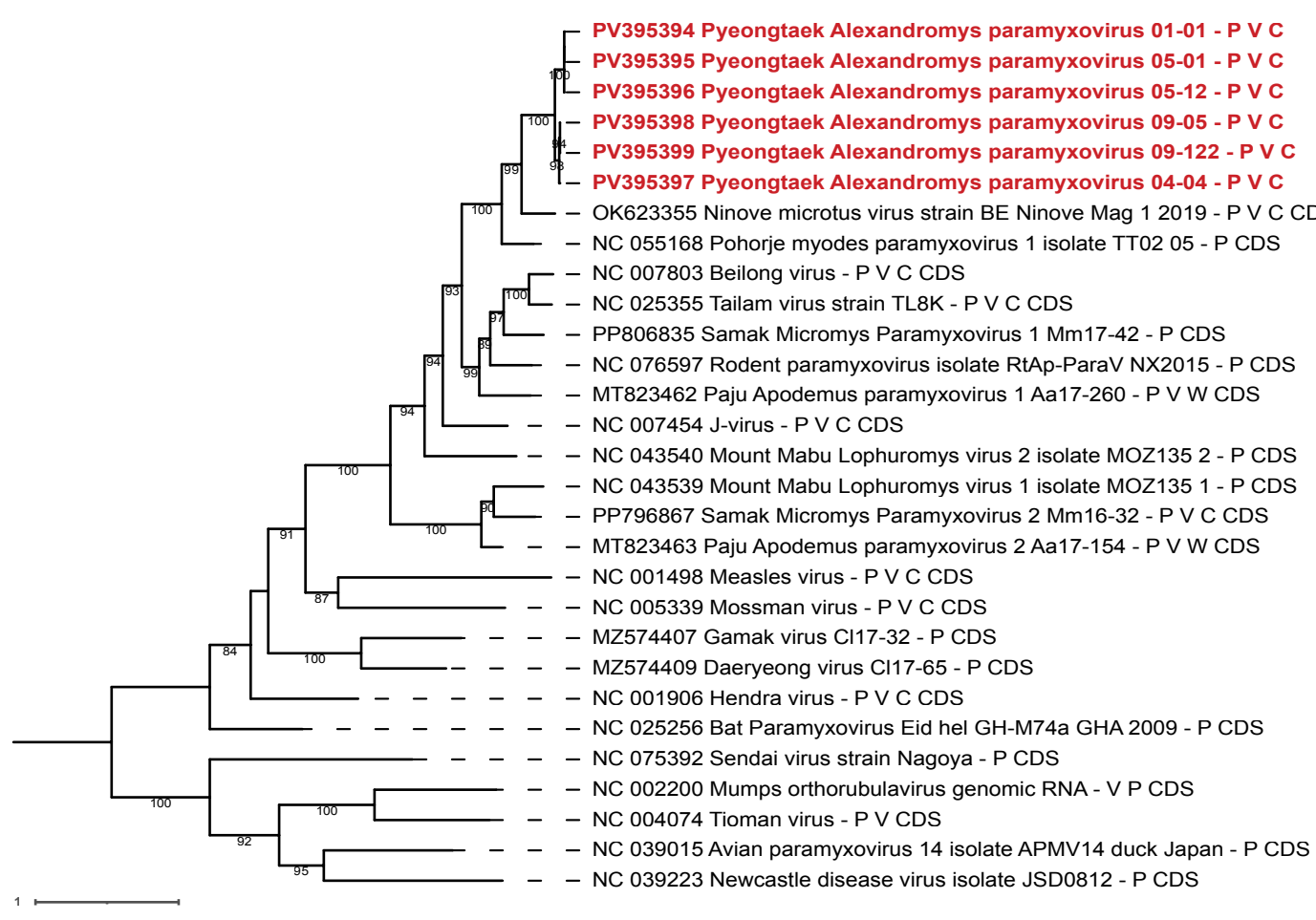

C)

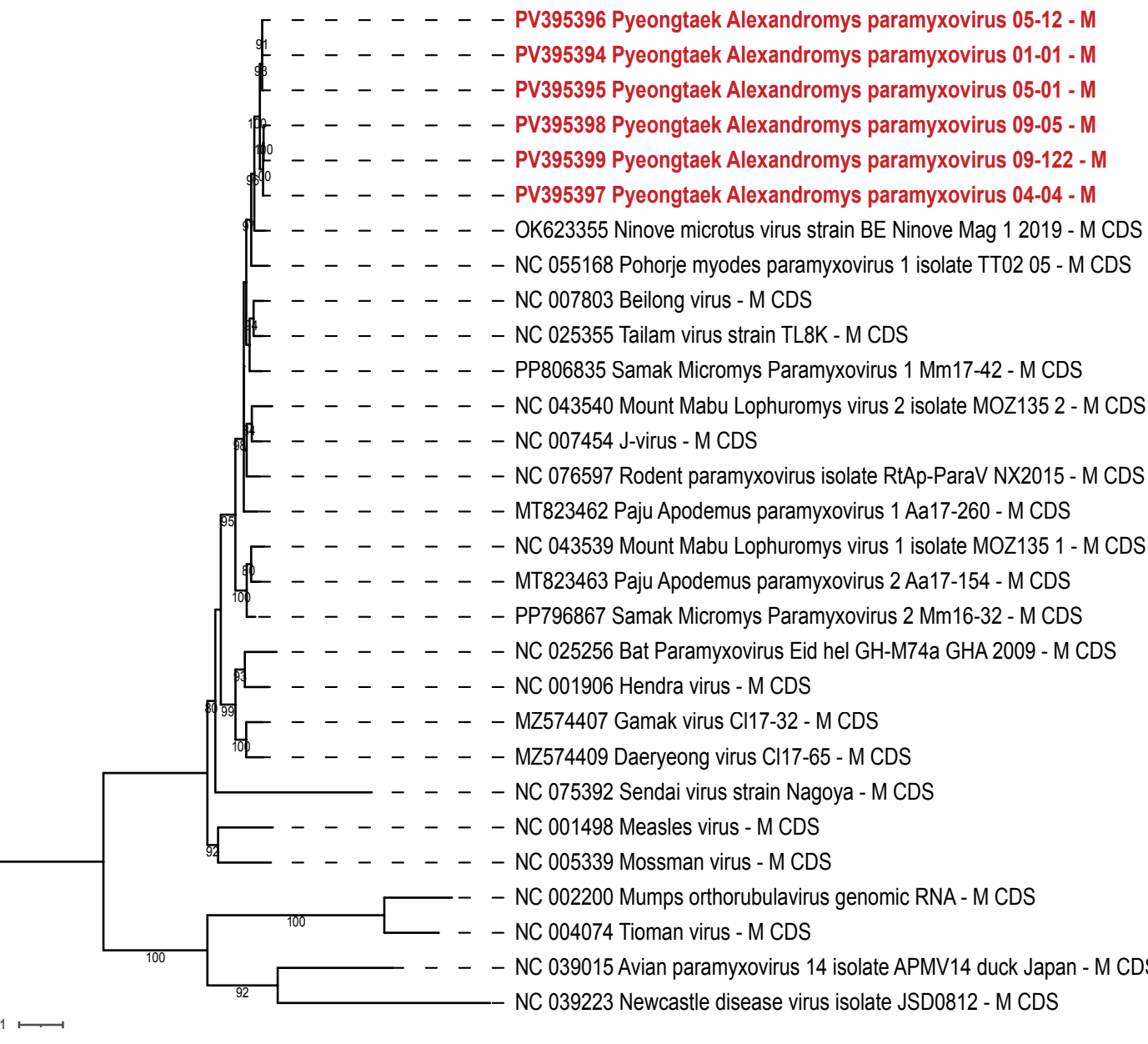

D)

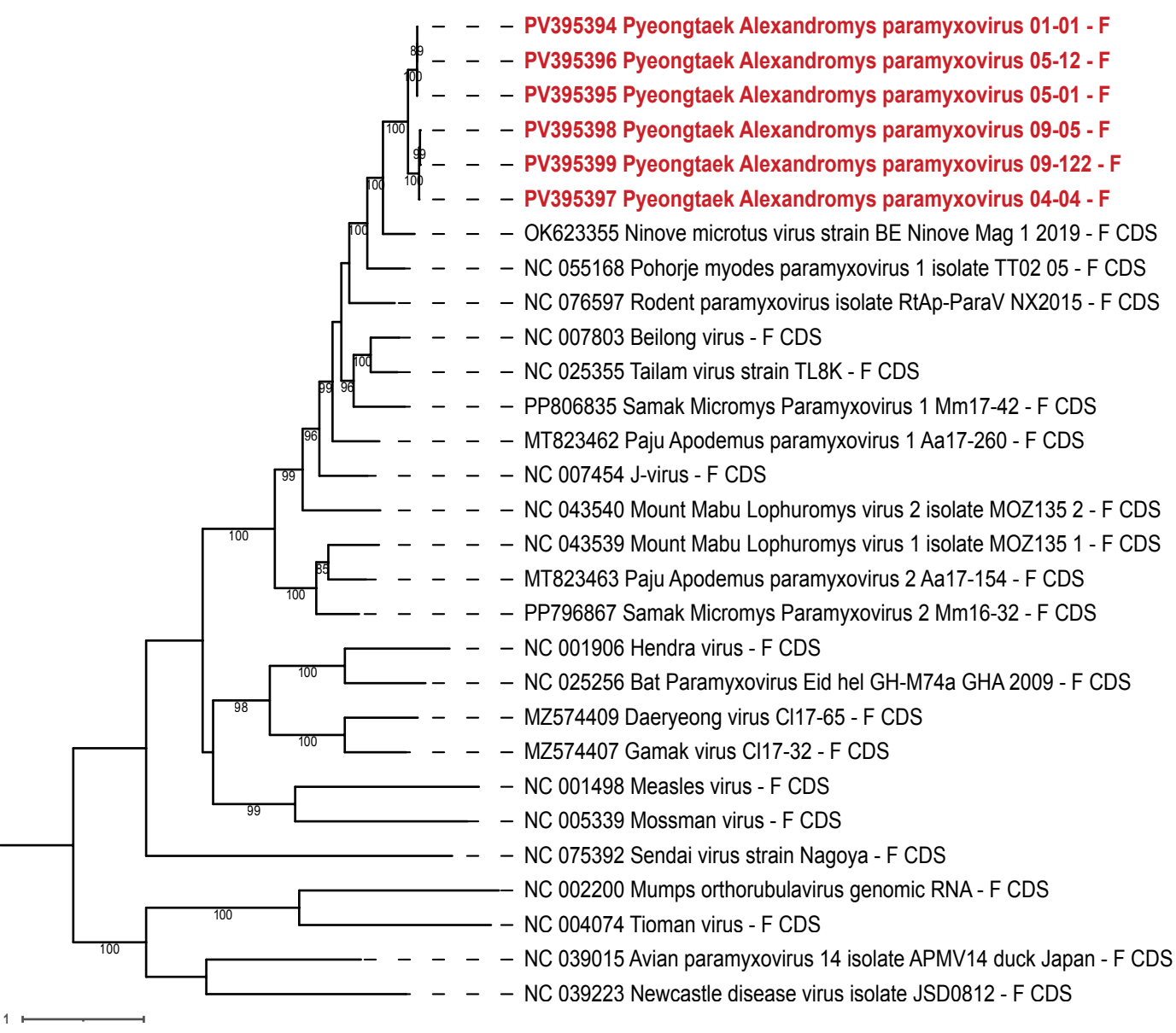

E)

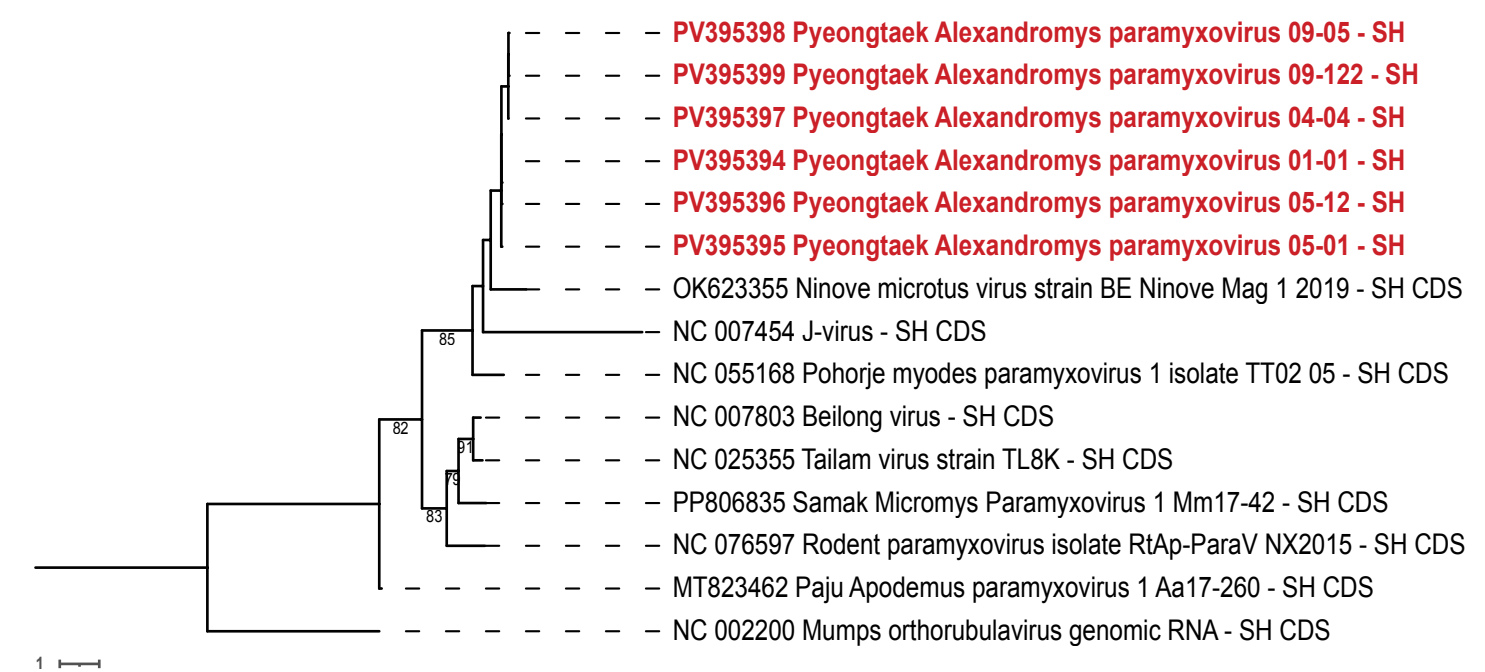

F)

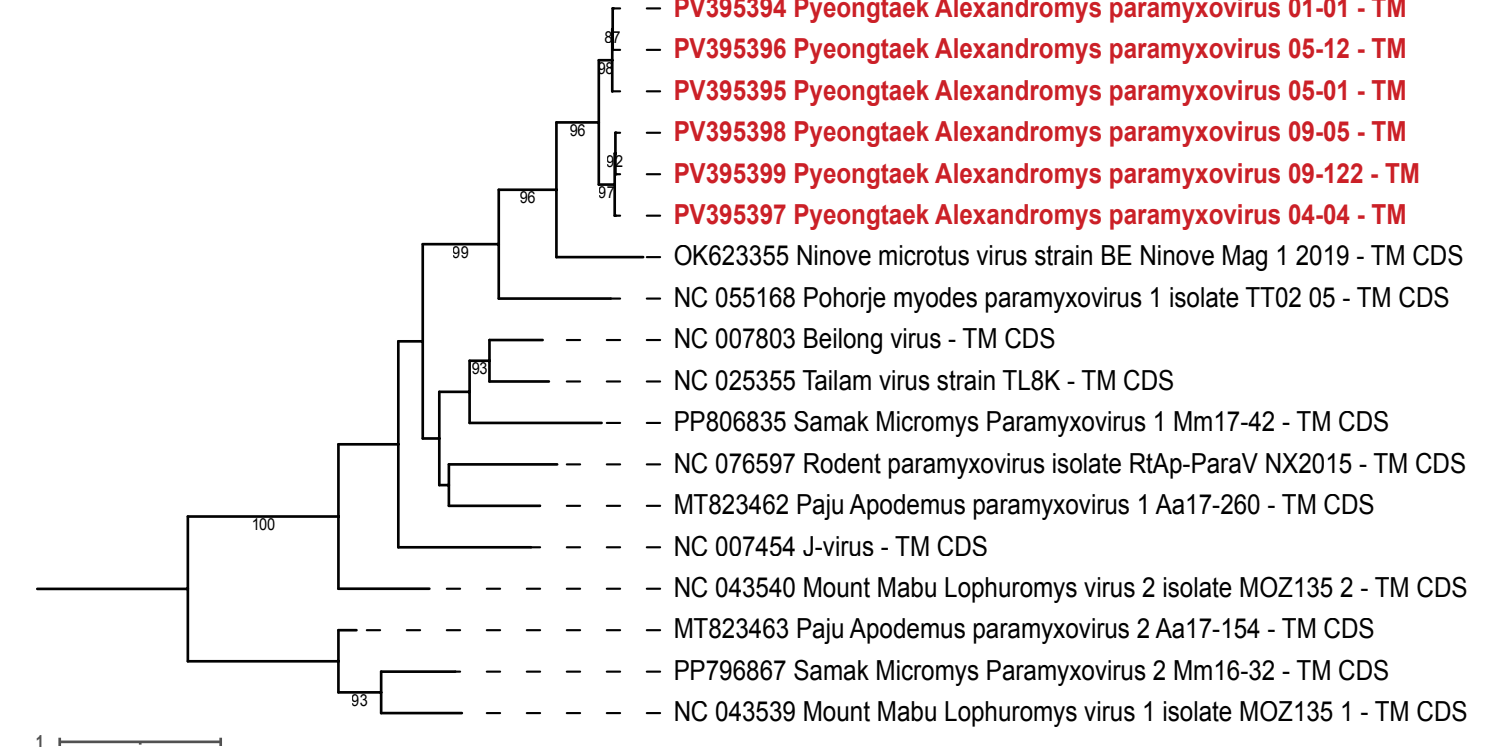

G)

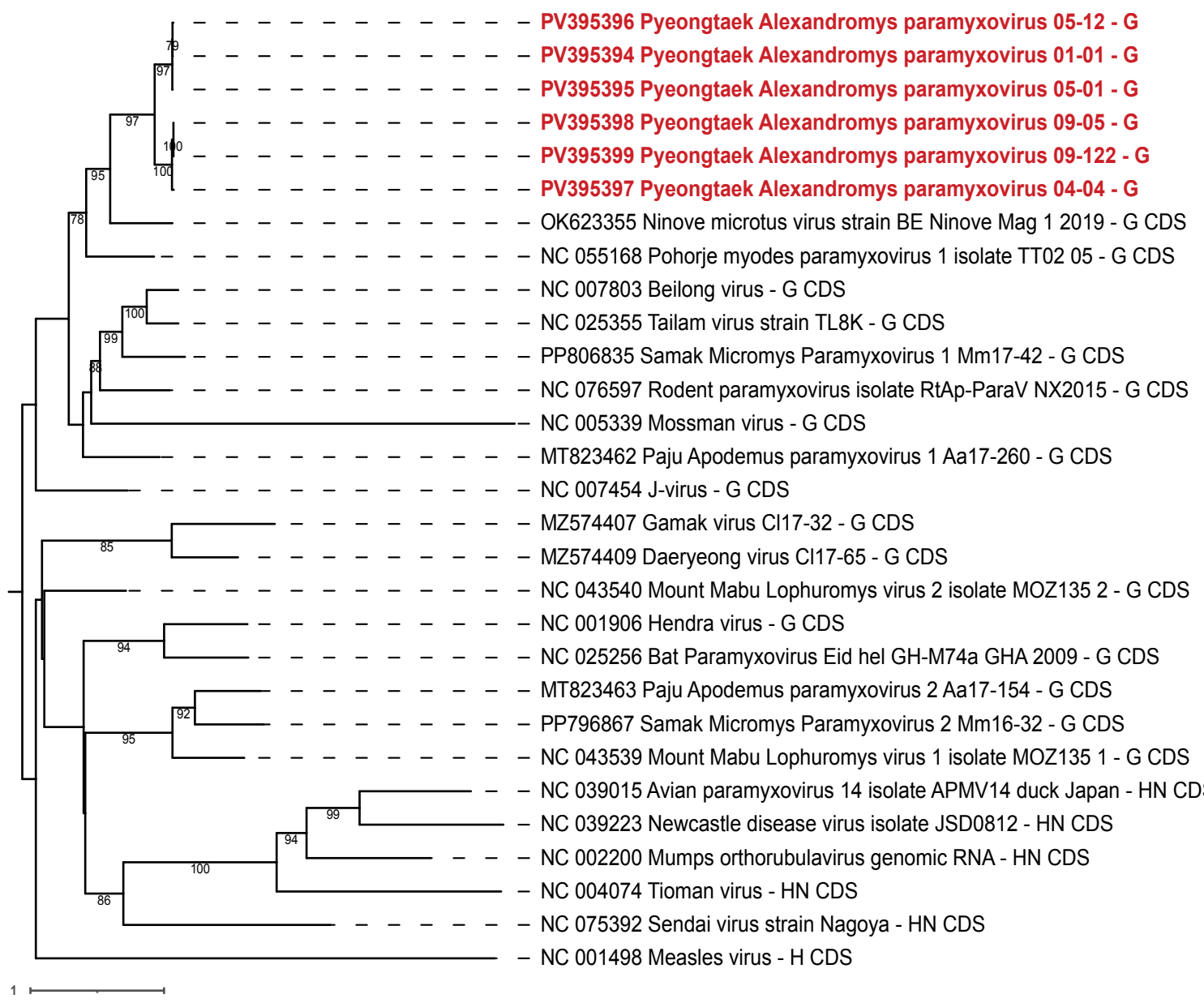

H)

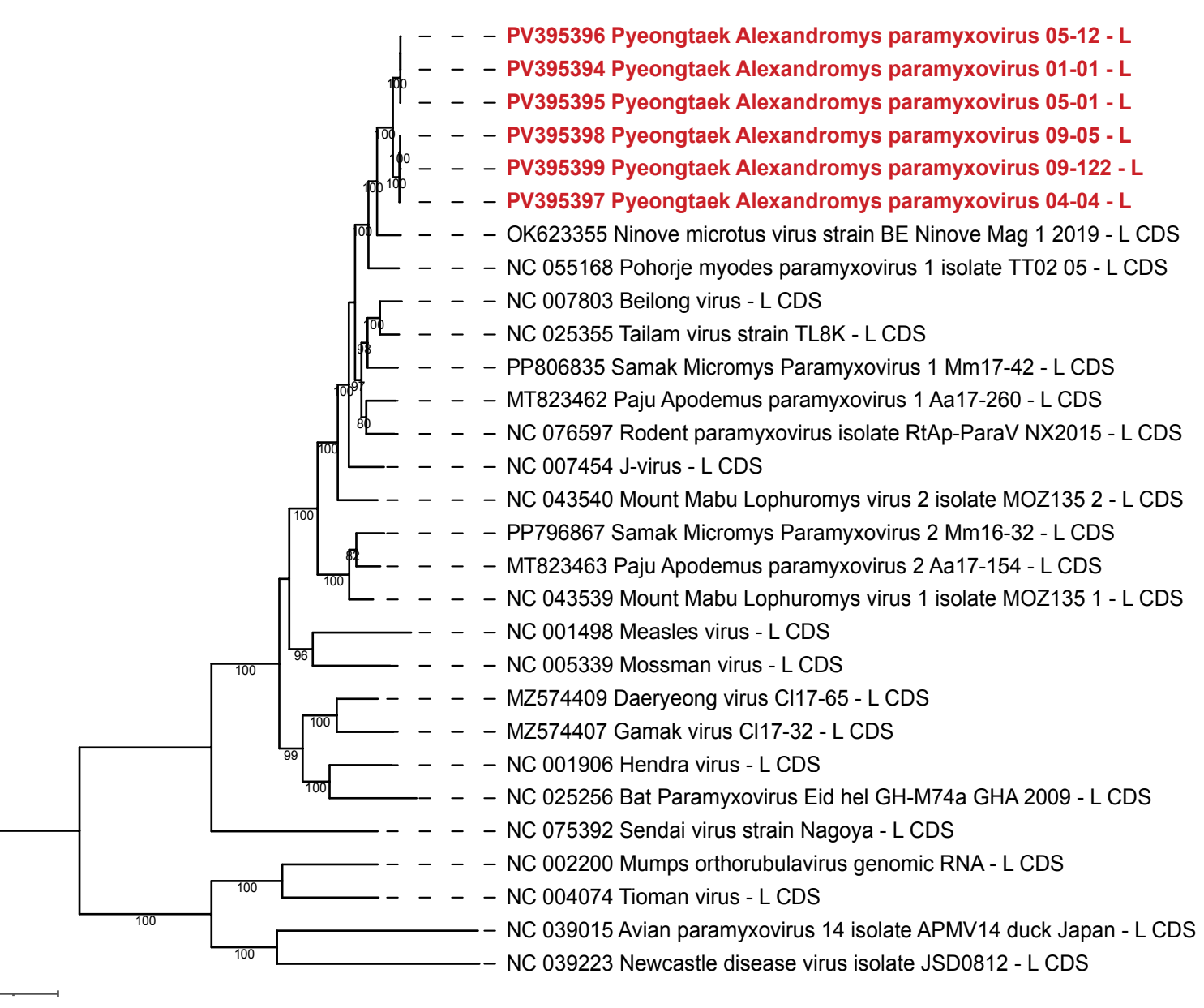

Supplement: Supplementary file 3 — Additional file 3. Phylogenetic tree of Pyeongtaek Alexandromys paramyxovirus (PyAPV) partial genomes with other paramyxoviruses. The phylogenetic tree was constructed using maximum likelihood analysis by IQTREE web server, employing the TPM3u+F+G4 model selected on the basis of BIC and incorporating 1000 bootstrapping iterations. The partial sequences generated two separate clusters, aligning with the phylogenetic tree derived from the complete genome sequences. N, nucleocapsid protein (A); P, phosphoprotein (B); M, matrix protein (C); F, fusion protein (D); SH, small hydrophobic protein (E); TM, transmembrane protein (F); G, glycoprotein; H, hemagglutinin protein; HN, hemagglutinin-neuraminidase protein (G); L, large protein (H). [file 13567_2026_1777_MOESM3_ESM.pdf]

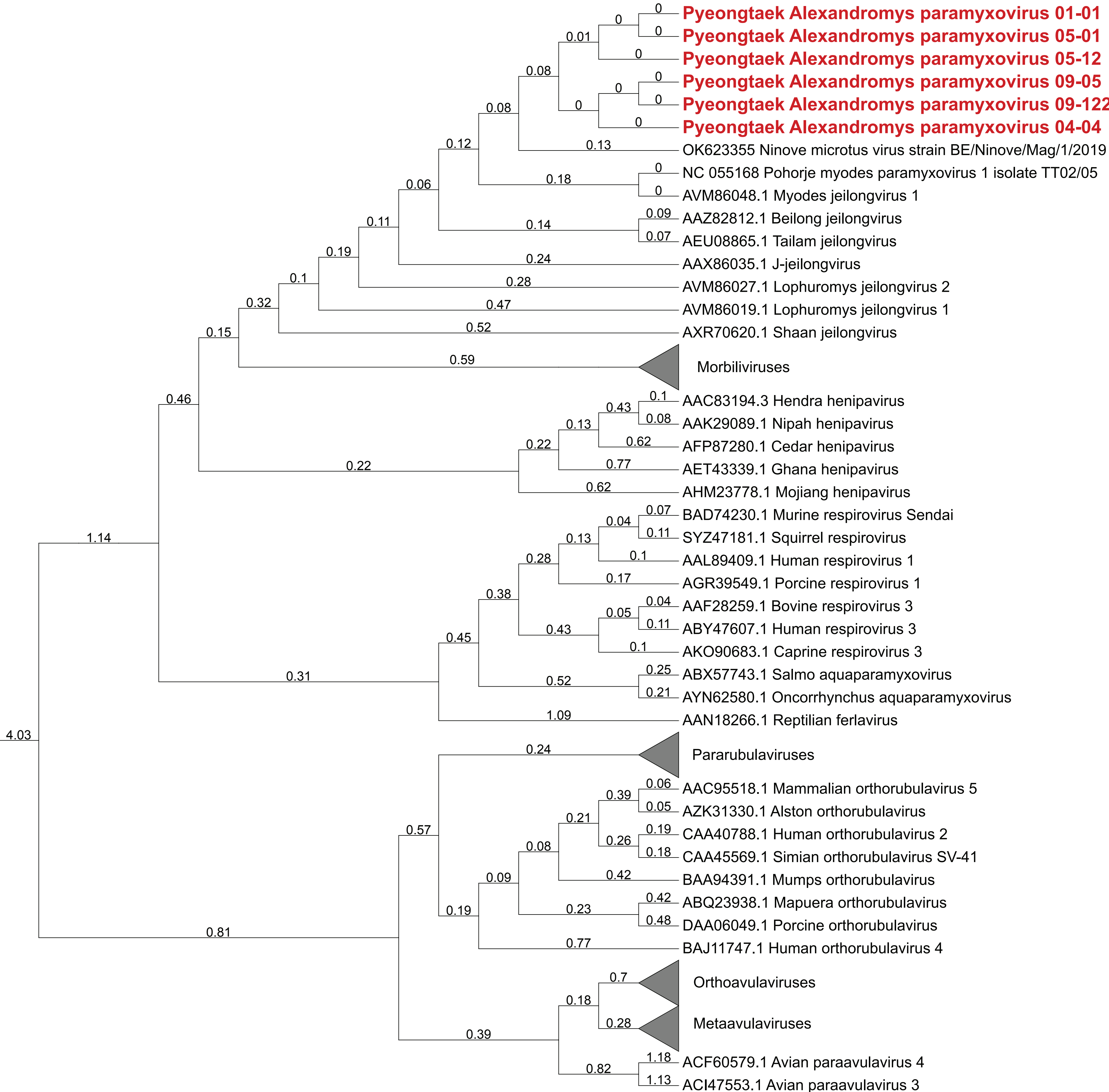

Supplement: Supplementary file 10 — Additional file 10. L Protein Phylogeny and Species Demarcation of Pyeongtaek Alexandromys paramyxovirus (PyAPV). The phylogenetic tree was constructed on the basis of amino acid sequences of the L protein, using the alignment template provided by the ICTV for species demarcation within the Paramyxoviridae family. Branch lengths are indicated on each branch. PyAPV sequences identified in this study are highlighted in red. GenBank accession numbers of reference viruses are shown on the right of taxon names. [file 13567_2026_1777_MOESM10_ESM.pdf]

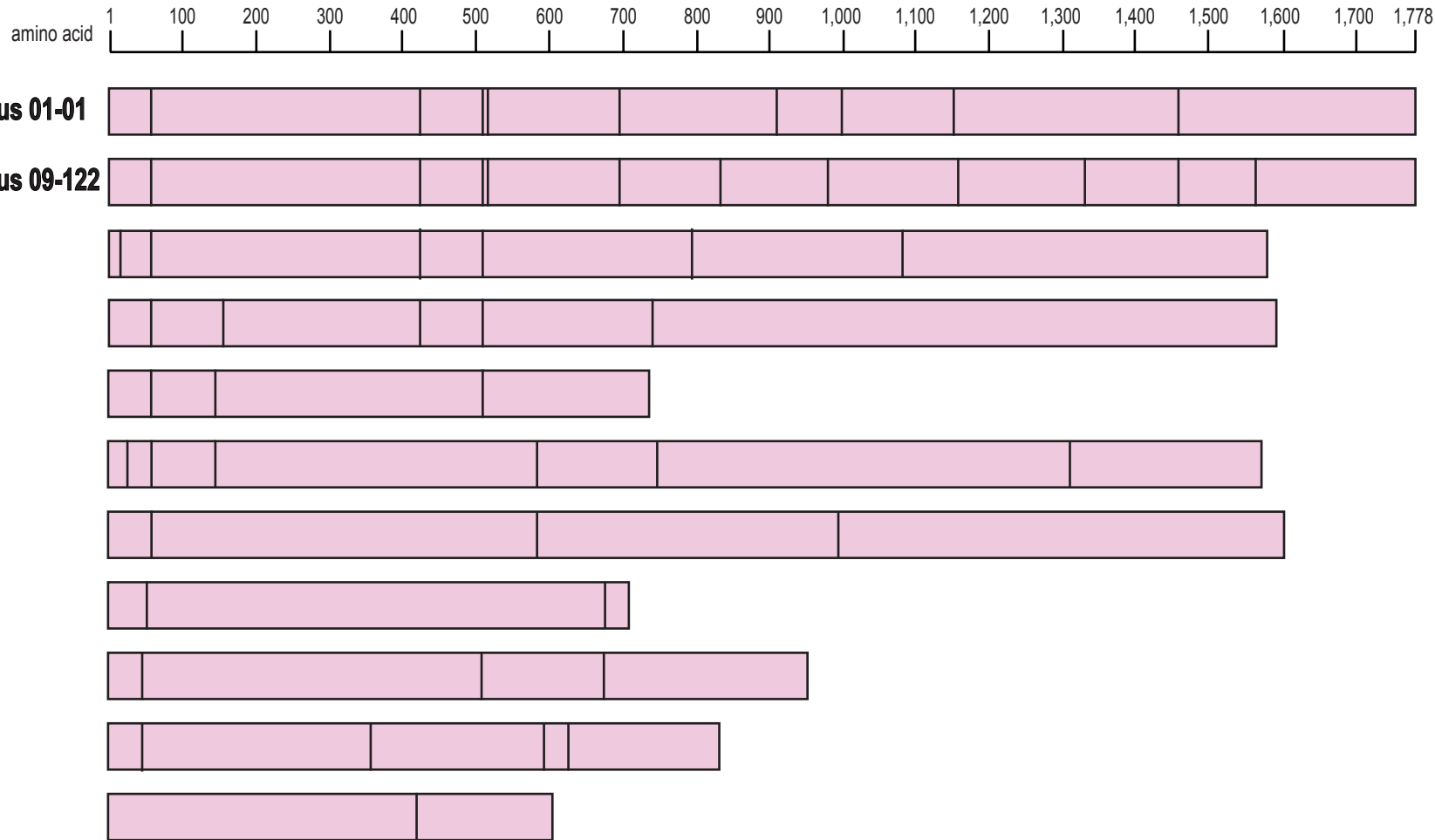

Supplement: Supplementary file 12 — Additional file 12. The predicted N-glycosylation site of PyAPV glycoproteins in comparison with representative Paramyxoviridae members. Multiple sequence alignment of full-length glycoproteins from ten Jeilongvirus members and one Henipavirus reference. N-linked glycosylation sites predicted with 9/9 consensus (“+” calls only) are indicated by vertical markers within pink bars representing glycoprotein length. [file 13567_2026_1777_MOESM12_ESM.pdf]
